# Influence of the surface flaws in oil-tempered wires on the fatigue life of automotive engine valve springs

**DOI:** 10.1038/s41598-022-25597-1

**Published:** 2022-12-07

**Authors:** Dae-Cheol Ko, Nam-Sik Ahn, Kyung-Hun Lee

**Affiliations:** 1 Department of Nanomechatronics Engineering, Pusan National University, Busan, 46241 Republic of Korea; 2 Product Engineering, ERAE AMS Co., Ltd., Daegu, 42981 Republic of Korea; 3 Division of Coast Guard Studies, Korea Maritime and Ocean University, Busan, 49112 Republic of Korea

**Keywords:** Engineering, Materials science

## Abstract

The purpose of this study is to evaluate the fatigue life of an automotive engine valve spring when the micro defect is applied to a 2300 MPa-class oil-tempered wire (OT wire) with 2.5 mm of diameter as the critical flaw depth. First, the deformation of the surface flaws in the OT wire during the valve spring manufacturing processes was derived via FE analysis using the sub-modeling technique, and the residual stress of the final spring was measured and applied to the spring stress analysis model. Second, the strength of the valve spring was analyzed to examine the presence of residual stress and compare the applied stress levels by the surface flaw. Third, the influence of micro defects on the fatigue life of the spring was evaluated by applying the stress on the surface flaw derived through the spring strength analysis to the S–N curve derived through a rotary bending fatigue test with the OT wire. The flaw depth of 40 µm, which is the existing criterion for surface flaw management, does not reduce the fatigue life.

## Introduction

Lightweight automotive parts are in great demand in the automotive industry to improve the fuel efficiency of motor vehicles. Consequently, the application of advanced high-strength steel (AHSS) has been increasing in recent years. An automotive engine valve spring mainly comprises an oil-tempered wire (OT wire) with high heat resistance, fatigue resistance, and sag resistance.

The OT wires currently in use aid in reducing the size and weight of engine valve springs owing to their high tensile strength (1900–2100 MPa); they can improve fuel efficiency by reducing friction with surrounding parts^1^. Because of these advantages, the use of high-tension wires has been rapidly increasing, and 2300 MPa-class ultra-high-strength wires have been developed. Long fatigue life is desired for automotive engine valve springs because they work under high levels of cyclic stresses. To satisfy this requirement, manufacturers generally design valve springs considering a fatigue life of greater than 5.5 × 10^7^ cycles and apply residual stress to the surface of the valve springs through the shot peening and hot setting processes to improve their fatigue life^2^.

Various research on the fatigue life of the automotive coil spring in the conventional operating environment has been sufficiently conducted. Gzal et al. presented an analytical, experimental, and finite element (FE) analysis of an elliptical cross-section helical spring with a small helix angle under static load. This study provides an explicit and simple expression for the location of the maximum shear stress as a function of the aspect ratio and spring index and enables obtaining the maximum shear stress analytically i.e., a crucial parameter in terms of practical design^3^. Pastorcic et al. described the failure and fatigue analysis results of a coil spring removed from a personal vehicle after having failed in service. Using experimental methods, the fractured spring was examined, and from the results, it can be concluded that this is an example of corrosion fatigue failure^4^. Kong et al. developed multiple linear regression-based spring durability models for fatigue life evaluation of automotive coil springs^5^. Putra et al. determined the life of an automotive coil spring due to road surface roughness. However, there have been few studies on how the surface flaw defects generated during the manufacturing process affect the life of the automotive coil spring^6^.

Surface flaws generated during the manufacturing processes cause local stress concentration in valve springs, thereby significantly reducing their fatigue life. The surface flaws on valve springs are caused by various factors, such as surface flaws in the raw materials used, defects in tools, and careless handling during the cold coiling process^7^. The surface flaws in raw materials are V-shaped with a steep slope, because of the hot rolling and multi-pass drawing processes, whereas the flaws caused by forming tools and careless handling exhibit a “U” shape with a gentle slope^8–11^. V-shaped flaws cause higher stress concentrations than U-shaped flaws; therefore, stringent flaw management standards are typically applied to the initial materials.

The current surface flaw management standards for OT wires include ASTM A877/A877M-10, DIN EN 10270-2, JIS G 3561, and KS D 3580. The DIN EN 10270-2 standard mandates the depth of surface flaws on wires of diameter 0.5–10 mm to be less than 0.5–1% of the wire diameter. Further, the JIS G 3561 and KS D 3580 standards mandate the depth of surface flaws on wires of diameter 0.5–8 mm to be less than 0.5% of the wire diameter. In the ASTM A877/A877M-10 standard, the manufacturer and buyer must mutually agree on the allowable surface flaw depth. To measure the depth of surface flaws on a wire, typically, the wire is corroded with hydrochloric acid, and the depths of flaws are measured using a micrometer. However, this method can measure flaws only in specific areas and not on the entire surface of the final product. Therefore, manufacturers use eddy current tests during the wire-drawing process to measure the surface flaws on continuously produced wires; these tests can measure surface flaw depths of up to 40 µm. The 2300 MPa-class wires under development have higher tensile strength and lower elongation than the existing wires whose tensile strengths have a range of 1900–2200 MPa; thus, it is believed that the fatigue life of the valve springs is very sensitive to surface flaws. Therefore, the safety of applying the surface flaw depth management standards of the existing 1900–2200 MPa-class wires to 2300 MPa-class wires must be verified.

The purpose of this study is to evaluate the fatigue life of an automotive engine valve spring when the minimum flaw depth measurable by eddy current tests (i.e., 40 µm) is applied to a 2300 MPa-class OT wire (diameter: 2.5 mm) as the critical flaw depth. The contributions and methods of this study are as follows.

1. A V-type flaw, which critically affects the fatigue life, was applied as the initial flaw in the OT wire, in the transverse direction concerning the axial direction of the wire. The aspect ratio (*α*) and length ratio (*β*) of the surface flaw were considered to observe the influence of its depth (*h*), width (*w*), and length (*l*). The surface flaw was applied to the inside of the spring, where fractures mainly occur.
2. To predict the deformation of the initial flaw in the OT wire during the cold coiling process, the sub-modeling method was applied, in which the analysis time and size of the surface flaw were considered because the flaw was very small compared with that in the global model.
3. The compressive residual stress in the spring after the two-stage shot peening process was predicted via FE analysis; the results were compared with those obtained through measurements after the shot peening process to validate the analysis model. Furthermore, the residual stress in the valve spring that underwent all the manufacturing processes was measured and applied in the strength analysis of the spring.
4. The stress in the surface flaw was predicted by analyzing the spring strength considering the deformation of the flaw during the cold coiling process and the compressive residual stress in the final spring.
5. A rotary bending fatigue test was conducted using an OT wire made of the same material as the valve spring. To assign the residual stress and the characteristics of the surface roughness of the fabricated valve spring to the OT wire, an S–N curve was derived through the rotary bending fatigue test after applying two-stage shot peening and torsion as pretreatment processes.
6. The fatigue life of the valve spring was predicted by applying the strength analysis results of the spring to the Goodman equation and S–N curve, and the effect of the surface flaw depth on the fatigue life was evaluated.

## Mechanical properties of the OT wire

A 2300 MPa-class OT wire having a diameter of 2.5 mm was used in this study to evaluate the fatigue life of an automotive engine valve spring. First, this wire was subjected to a tensile test, and its ductile fracture model was then obtained.

### Tensile test

**Figure 1:**
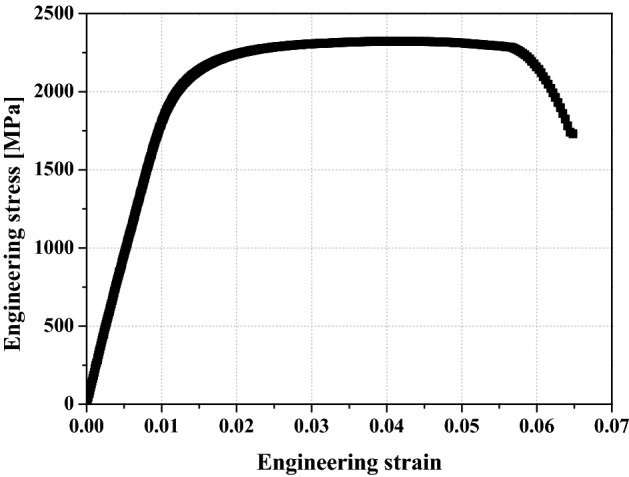
Engineering stress–strain curve of OT wire.

The mechanical properties of the OT wire were derived through a tensile test before performing an FE analysis on the cold coiling process and spring strength. The result of tensile tests under the strain rate of 0.001 s^−1^ is used for determining the material’s stress–strain curves, as shown in Fig. 1. The wire material used is SWONB-V and its yield stress, ultimate tensile stress, modulus of elasticity and Poisson’s ratio are 2001.2 MPa, 2316 MPa, 206 GPa, and 0.3, respectively. The flow stress–strain relationship was obtained as follows:

1 $$\overline{\sigma }=2806.7{\overline{\epsilon }}^{0.0646} \, \mathrm{MPa}.$$

### Ductile fracture model

**Figure 2:**
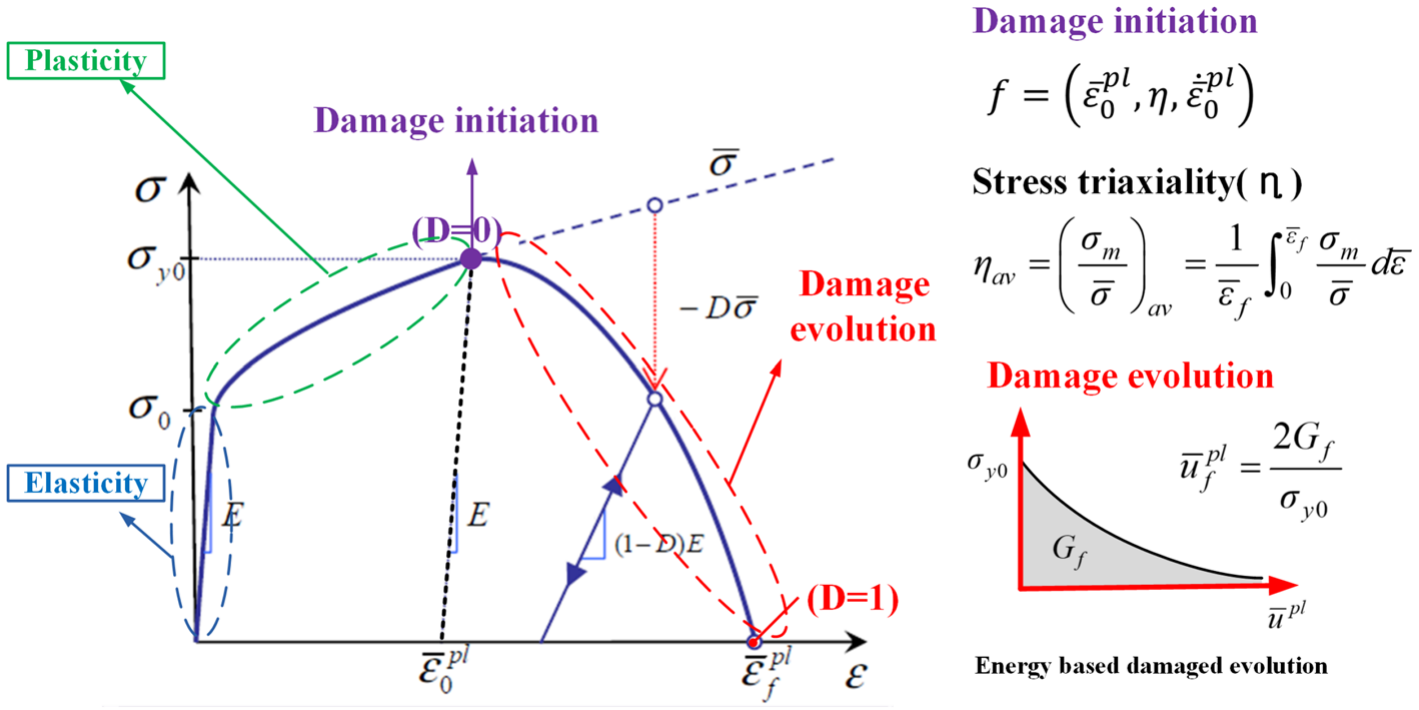
Schematic illustration of elastic–plastic material with progressive damage.

Figure 2 illustrates the process of ductile fracture. An elastic–plastic deformation occurs during the deformation of the material, and the necking of the material occurs when the stress in the material reaches its ultimate tensile strength. Subsequently, the generation, growth, and combination of voids inside the material result in the fracture of the material.

Stress-modified critical strain model that considers the influence of stress was used as the ductile fracture model, and the damage accumulation method was used for the fracture after necking. Here, damage initiation is expressed as a function of strain, stress triaxiality, and strain rate. The stress triaxiality is defined as the average value obtained by dividing the hydrostatic stress due to the material deformation until the time of necking by the effective stress. In the damage accumulation method, a fracture occurs when the damage value reaches 1, and the energy required to reach the damage value of 1 is defined as the fracture energy (*G*_*f*_). The fracture energy corresponds to the area from necking to the time of fracture in the true stress–displacement curve of the material.

**Figure 3:**
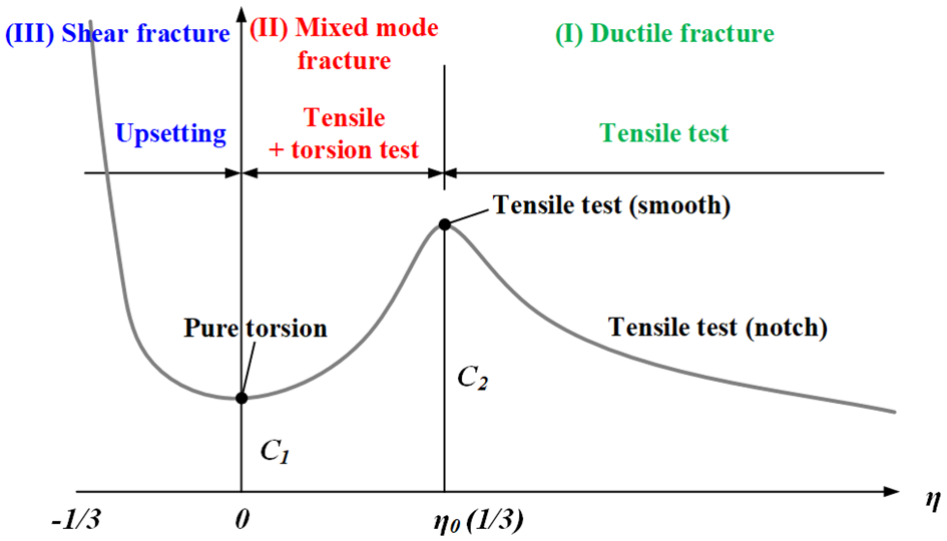
Three-branch fracture initiation locus in industrial steel.

In the case of conventional steel, depending on the stress mode, ductile fracture, shear fracture, or mixed-mode fracture caused by ductile and shear fractures occurs, as shown in Fig. 3. The fracture strain and stress triaxiality exhibit different values for each fracture mode.

In the region corresponding to stress triaxiality of greater than 1/3 (region I), ductile failure occurs, and the fracture strain and stress triaxiality can be derived through a tensile test on the specimens with no surface flaws and those with notches. In the region corresponding to stress triaxiality of 0 to 1/3 (region II), a combination of ductile and shear fractures occurs (i.e., mixed-mode fracture), and the fracture strain and stress triaxiality can be derived through a torsion test along with the application of tension. In the region corresponding to stress triaxiality of − 1/3 to 0 (III), shear fracture caused by compression occurs, and the fracture strain and stress triaxiality can be derived through an upsetting test.

In the case of OT wires used to manufacture engine valve springs, fractures caused by various stress modes must be considered in the manufacturing process and under application conditions. Thus, tensile and torsion tests were conducted to apply the fracture strain criteria, considering the effect of stress triaxiality on each stress mode, and a large-strain elastic–plastic FE analysis was performed to quantify the change in stress triaxiality. The compression mode was not considered because of the limitations in specimen processing, i.e., the diameter of the OT wire was only 2.5 mm. Table 1 summarizes the conditions for the tensile and torsion tests, as well as the stress triaxiality and fracture strain derived through FE analysis.

**Table 1:** Conditions and results of tensile and twist tests.

Test	Case	Tensile force (N)	Stress triaxiality, *η*	Fracture strain, *ε*_*f*_
Tensile + twist	B1	10	0.0265	0.0016
	B2	85	0.0751	0.0023
	B3	200	0.1083	0.0041
	B4	300	0.1343	0.0067
	C1	10	0.0279	0.0090
	C2	85	0.0794	0.0243
	C3	200	0.1200	0.0053
Tensile	Smooth	–	0.3333	0.0646
	Notch R0.8		0.5780	0.0303
	Notch R0.4		0.5969	0.0265

The fracture strain of conventional steel for stress triaxiality can be predicted using the following equation.

2 $$\overline{{\varepsilon _{0} }} ^{{pl}} = \left\{ {\begin{array}{*{20}l} {\infty ,} & {\eta \le - 1/3} \\ {C_{1} /(1 + 3\eta ),} & { - 1/3 < \eta \le 0} \\ {C_{1} + (C2 - C1)(\eta /\eta _{0} )^{2} ,} & {0 \le \eta \le \eta _{0} } \\ {C_{2} \eta _{0} /\eta ,} & {\eta _{0} \le \eta } \\ \end{array} ,} \right.$$

where *C*_1_: ${\overline{{\varepsilon }_{0}}}^{pl}$ pure shear (*η* = 0), and *C*_2_: ${\overline{{\varepsilon }_{0}}}^{pl}$ uniaxial tension (*η* = *η*_0_ = 1/3).

**Figure 4:**
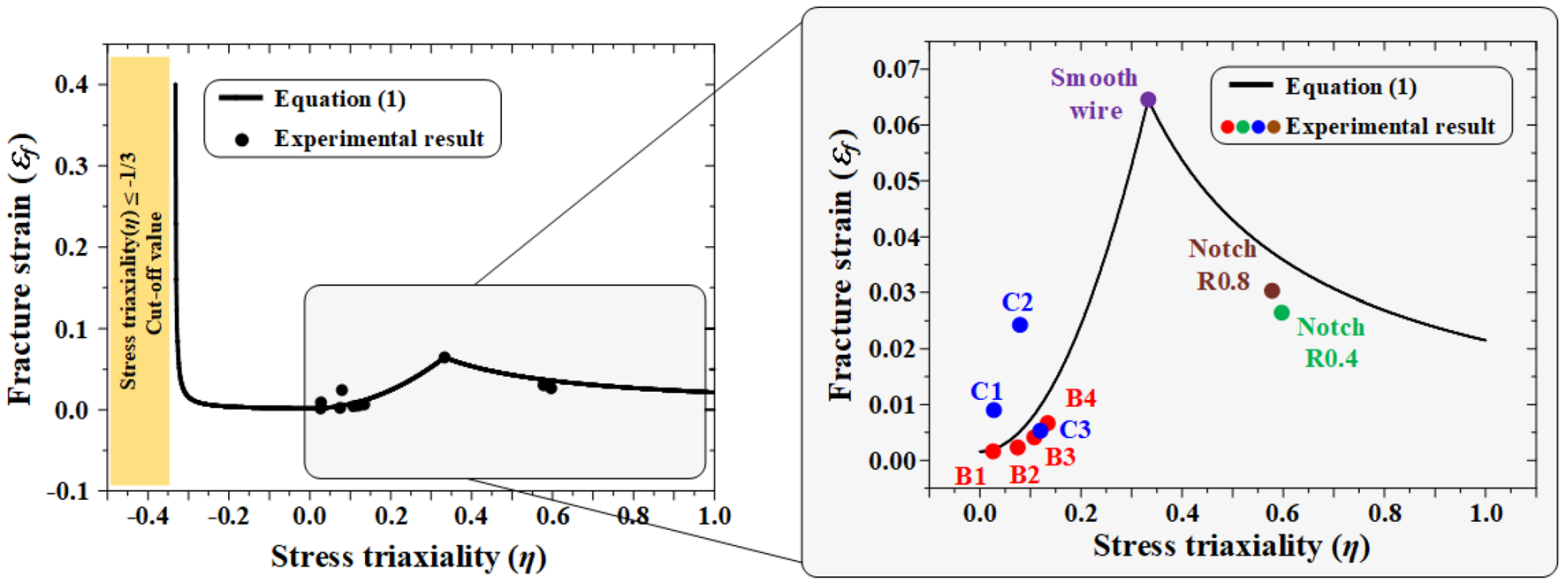
Fracture strain as a function of stress triaxiality.

The trend lines for each stress mode were derived by applying the values of the fracture strains *C*_*1*_ and *C*_*2*_ in Eq. (2); *C*_*1*_ and *C*_*2*_ were derived from tensile and torsion tests using specimens with no surface flaws. Figure 4 shows the stress triaxiality and fracture strain values derived through the tests and the trend line predicted using Eq. (2). The trend line and the relationship between the stress triaxiality and fracture strain derived from the tests exhibited similar tendencies. The fracture strain and stress triaxiality for each stress mode derived by the application of trend line was applied as the criteria for ductile fracture.

**Figure 5:**
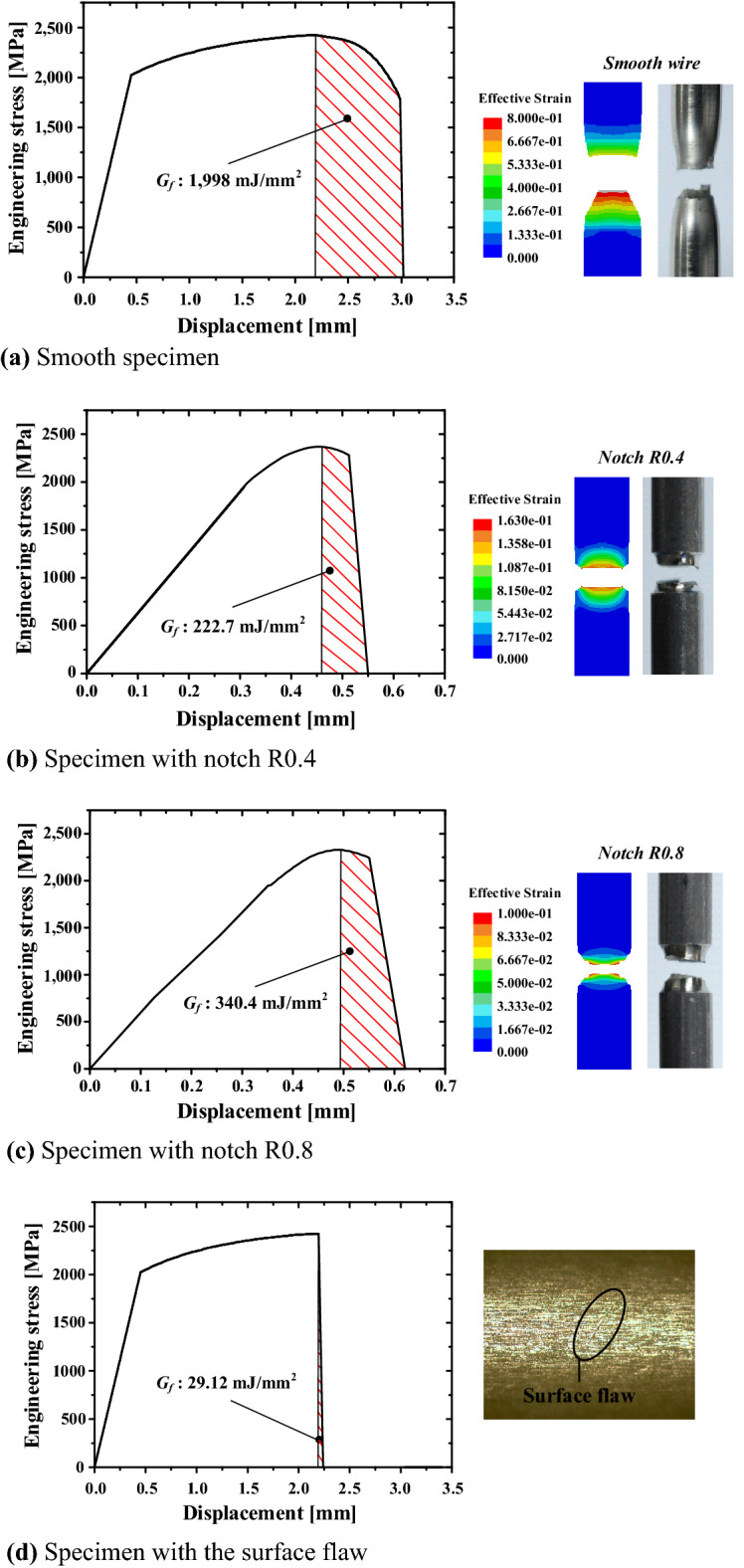
Fracture energy obtained through a tensile test and FE analysis.

The fracture energy was applied as a material property that determines the time of fracture after the necking of the material and can be derived through a tensile test. The fracture energy varies depending on the presence or absence of flaws on the material surface because the time of fracture varies depending on the local stress concentration. Figure 5a–c show the fracture energy of a specimen without surface flaws and specimens with notch R0.4 or R0.8 derived from tensile tests and FE analysis. The fracture energy corresponds to the area from necking to the time of fracture in a true stress–displacement curve.

The fracture energy of an OT wire with fine surface flaws was predicted through a tensile test on an OT wire with a flaw deeper than 40 µm, as shown in Fig. 5d. Ten specimens with a flaw were used in the tensile test, and the average fracture energy was evaluated to be 29.12 mJ/mm^2^.

## Standardization of surface flaws in the OT wire

**Figure 6:**
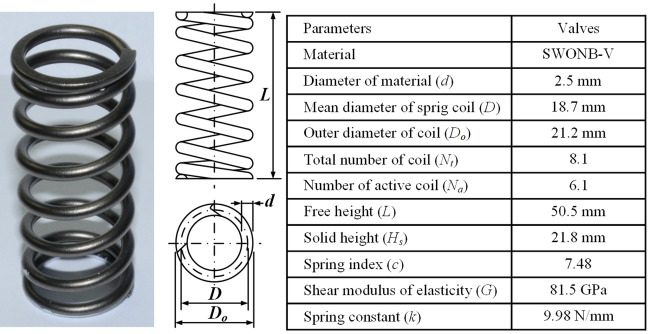
Dimensions of the automotive engine valve spring.

A standardized surface flaw is defined as the ratio of the flaw depth to the valve spring wire diameter, regardless of the surface flaw geometry of the OT wire used in the manufacture of automotive engine valve springs. The flaws in an OT wire can be classified based on the direction, geometry, and length. The stress levels acting on the flaws on the spring surface differ depending on the geometry and direction of the flaws even for the same flaw depth; thus, the geometry and direction of the flaws affect the fatigue strength. Therefore, the flaw geometry and direction that most critically affect the fatigue life of a spring must be considered to apply strict management criteria for surface flaws. The fatigue life of OT wires is very sensitive to notches owing to their fine-grained structure. Therefore, the flaw that exhibits the highest stress concentration according to the flaw geometry and direction must be set as the initial flaw through FE analysis. Fig. 6 shows the 2300 MPa-class ultrahigh-strength automotive valve spring used in this study.

### Classification of surface flaw position

The surface flaws in the OT wire were classified into the inside and outside flaws based on the spring axis. Owing to the bending during the cold coiling process, compressive stress, and tensile stress act on the inside and outside of the spring, respectively. A fracture could cause by a surface flaw that occurs on the outside due to tensile stress during the cold coiling process.

In actual application, springs are subjected to periodic compression and relaxation. During the compression of a spring, the wire is twisted and higher shear stress acts on the inside of the spring than on the surroundings due to stress concentration^7^. Thus, if a surface flaw exists on the inside of the spring, the possibility of spring fracture is the highest. Therefore, the outside of the spring (where the fracture is expected during the manufacture of the spring) and the inside (where the highest stress occurs during actual application) were set as the surface flaw positions.

### Classification of surface flaw geometry

The surface flaw geometry of the OT wire is classified into U-type, V-type, Y-type, and T-type. The Y- and T-types are mainly found in the surface flaws of raw materials, and the U- and V-type flaws are generated during the cold coiling process because of tools and careless handling. As for the surface flaw geometry of raw materials, the U-type flaws generated due to non-uniform plastic deformation during the hot rolling process deform into V-, Y-, and T-type seam defects during the multi-pass drawing process^8,10^.

In addition, the V-, Y-, and T-type flaws whose slope of the surface flaw notch is steep are subjected to high-stress concentration while the spring is in operation. The valve spring is subjected to bending during the cold coiling process and torsion during operation. The stress concentrations on the V- and Y-type flaws, which have relatively high-stress concentrations, were compared by FE analysis; ABAQUS, a commercial software, was used in the FE analysis. The stress–strain relationship is shown in Fig. 1 and Eq. (1). Two-dimensional (2D) rectangular four-node elements were used in this simulation, and the minimum edge length of elements is equal to 0.01 mm. As for the analysis models, V- and Y-type flaws with a depth of 0.5 mm and flaw inclination angle of 2° were applied to 2D models of a wire with a diameter of 2.5 mm and length of 7.5 mm.

**Figure 7:**
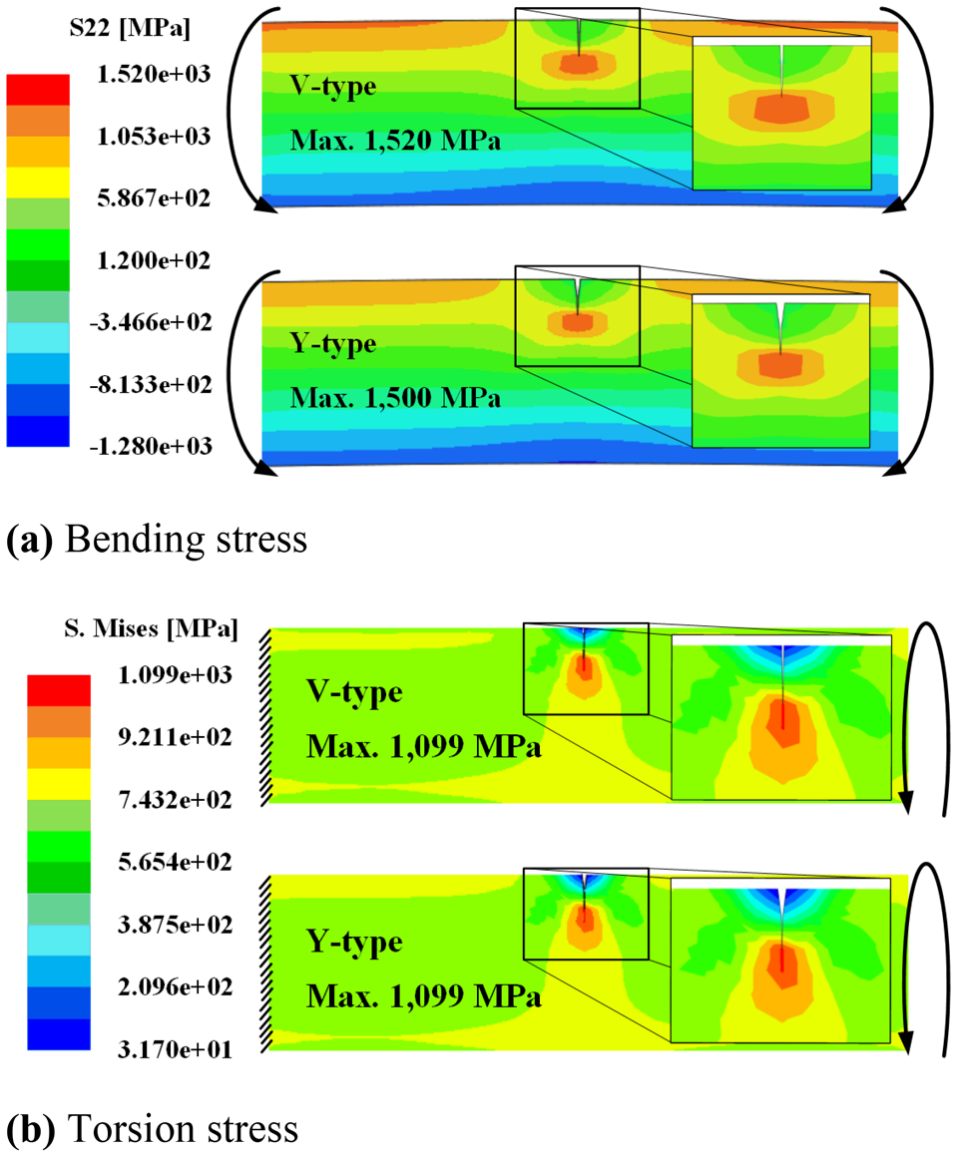
Effective stress distribution in a 2D model with Y-type and V-type surface flaws.

Figure 7a shows the stress concentration at the tip of each flaw caused by bending when a bending moment of 1500 N∙mm was applied at both ends of each wire. The analysis results showed that the maximum stresses of 1038.7 and 1025.8 MPa occurred at the tips of the V-type and Y-type flaws, respectively. Figure 7b shows the stress concentration at the tip of each flaw caused by torsion. When the left side was constrained and a torsional moment of 1500 N∙mm was applied to the right side, the same maximum stress of 1099 MPa occurred at the tips of both the V-type and Y-type flaws. These results indicate that the V-type flaw exhibits higher stress than the Y-type flaw for bending when they had the same depth and flaw inclination angle, but they exhibit the same stress for torsion. Therefore, the V- and Y-type surface flaws with the same depth and flaw inclination angle can be standardized as V-type, which has higher maximum stress caused by stress concentration. The aspect ratio of the V-type flaw is defined as α = w/h, using its depth (*h*) and width (*w*) for the V- and T-type flaws; therefore, the T-type flaw (*α* ≈ 0) geometry can be replaced with the V-type flaw geometry. Therefore, the Y- and T-type flaws can be standardized with a V-type flaw. The length ratio is additionally defined as *β* = *l/h*, using the depth (*h*) and length (*l*).

### Classification of surface flaw direction

**Figure 8:**
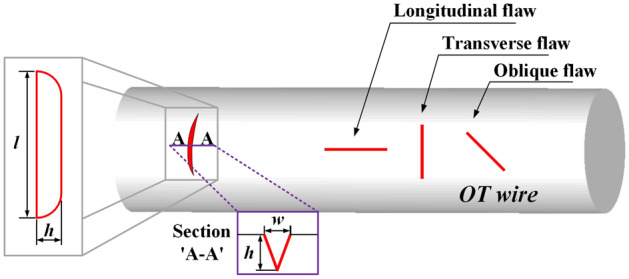
Schematic illustration and direction of surface flaws on the OT wire.

The directions of the surface flaws on the OT wire were classified into longitudinal, transverse, and oblique directions concerning the axial direction of the wire, as shown in Fig. 8^11^. The influence of the surface flaw direction on the spring strength was evaluated via FE analysis.

**Figure 9:**
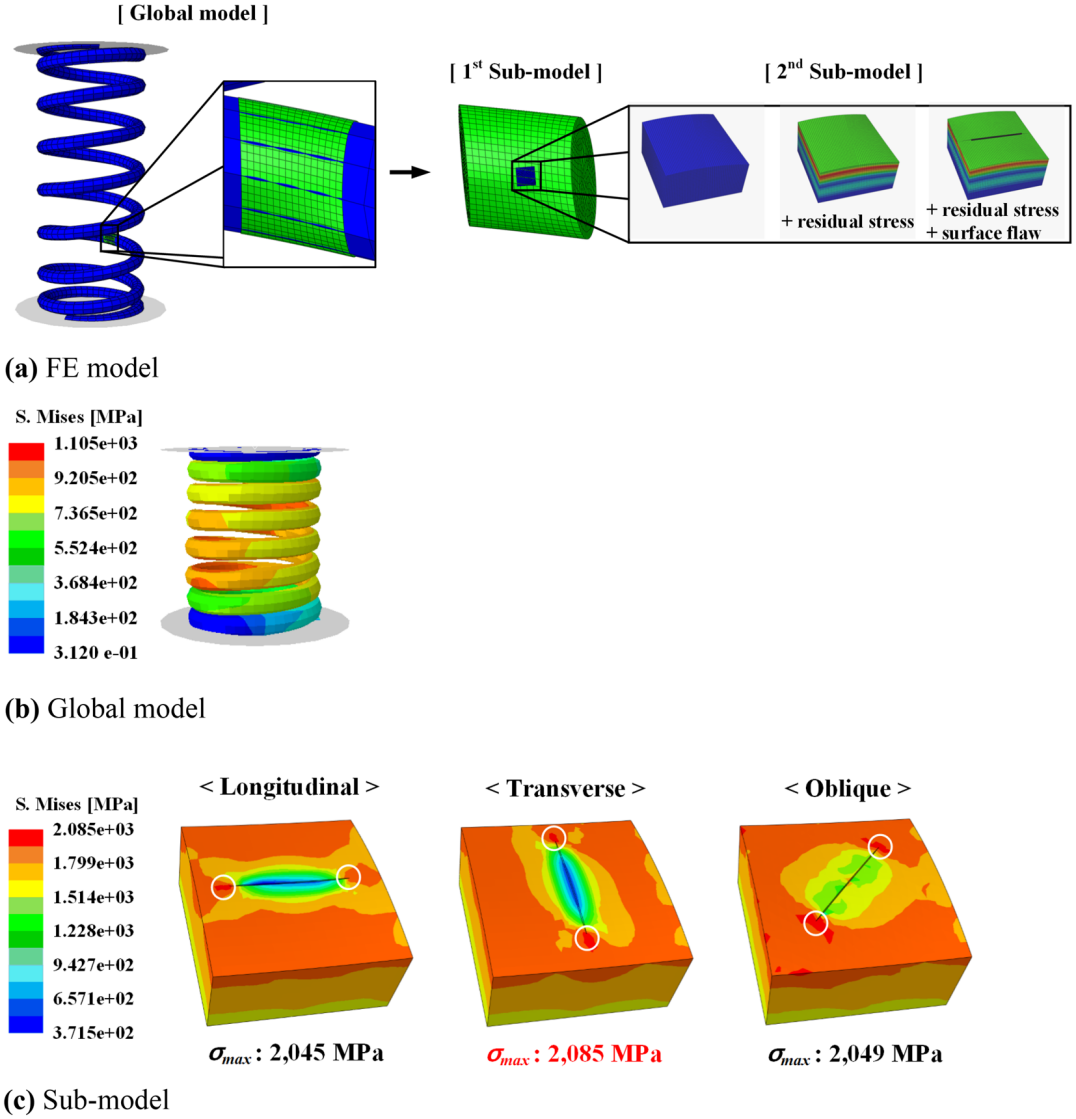
Stress analysis model and its result; effective stress distribution in a fully compressed spring.

Figure 9a shows the stress analysis model for the engine valve spring. As an analysis condition, the spring was compressed from its free height of 50.5 mm to its solid height of 21.8 mm; maximum stress of 1086 MPa occurred inside the spring, as shown in Fig. 9b. Because the fracture of an actual engine valve spring occurs mainly on the inside of the spring, the presence of a surface flaw on the inside is expected to critically affect the fatigue life of the spring. Therefore, surface flaws in the longitudinal, transverse, and oblique directions were applied to the inside of the engine valve spring using the sub-modeling technique. Table 2 lists the dimensions of the surface flaws and the maximum stress for each flaw direction under the maximum compression of the spring. The highest stress was observed in the transverse direction, and the stress ratio in the longitudinal direction and that of the oblique direction to the transverse direction were evaluated to be 0.934–0.996. The stress ratio is simply determined by dividing that value by the maximum transverse stress. The maximum stress in the spring occurred at the tip of each surface flaw, as shown in Fig. 9c. The stress values of 2045, 2085, and 2049 MPa were observed in the longitudinal, transverse, and oblique directions, respectively. These analysis results signify that transverse surface flaws have the most direct effect on the fatigue life of engine valve springs.

**Table 2:** Comparison of effective stress in various directions.

No. of case	Dimension (*h*/*w*/*l*) (µm)	Direction	Max. stress (MPa)	Stress ratio
1	40 / 8 / 1000	Longitudinal	1977	0.934
		Oblique	2026	0.958
		Transverse	2116	1
2	30 / 6 / 800	Longitudinal	1958	0.988
		Oblique	1973	0.996
		Transverse	1981	1
3	40 / 22 / 200	Longitudinal	2045	0.981
		Oblique	2049	0.983
		Transverse	2085	1

### Standardized surface flaw

The V-type flaw, which is expected to affect the fatigue life of the engine valve springs most directly, was selected as the initial flaw in the OT wire; the transverse direction was selected as the flaw direction. The flaw was applied not only to the outside of the engine valve spring for disconnection during manufacture, but also to the inside where the highest stress occurred due to stress concentration during operation. The maximum depth of the flaw was set to 40 µm, which could be detected by eddy current tests, and the depth corresponding to 0.1% of the wire diameter of 2.5 mm was set as the minimum depth. Thus, the depth of the flaw ranged between 2.5 and 40 µm. The depth, length, and width of the flaw for the aspect ratio of 0.1–1 and length ratio of 5–15 were set as variables, and their influence on the fatigue strength of the spring was evaluated. Table 3 summarizes the analysis conditions determined using the response surface method.

**Table 3:** Initial surface flaws in the OT wire.

No. of case	Depth (*h*) (µm)	Width (*w*) (µm)	Length (*l*) (µm)	Aspect ratio (*α* = *w/h*)	Length ratio (*β* = *l/h*)
1	40	22	600	0.55	15
2	2.5	0.25	25	0.1	10
3	40	22	200	0.55	5
4	2.5	2.5	25	1	10
5	40	4	400	0.1	10
6	21.25	11.69	212.5	0.55	10
7	40	40	400	1	10
8	2.5	1.38	37.5	0.55	15
9	21.25	2.12	106.25	0.1	5
10	21.25	21.25	318.75	1	15
11	2.5	1.38	12.5	0.55	5
12	21.25	2.13	318.75	0.1	15
13	21.25	21.25	106.25	1	5

## Flaw deformation after each spring manufacturing process

An automotive engine valve spring is manufactured through the cold coiling, tempering, shot peening, and hot setting processes of an OT wire. Changes in surface flaws during the spring manufacturing processes must be considered to evaluate the effect of the initial surface flaw in the OT wire on the fatigue life of the engine valve spring. Therefore, in this section, the deformation of the surface flaw in the OT wire in each spring manufacturing process is predicted using FE analysis.

### Cold coiling process

**Figure 10:**
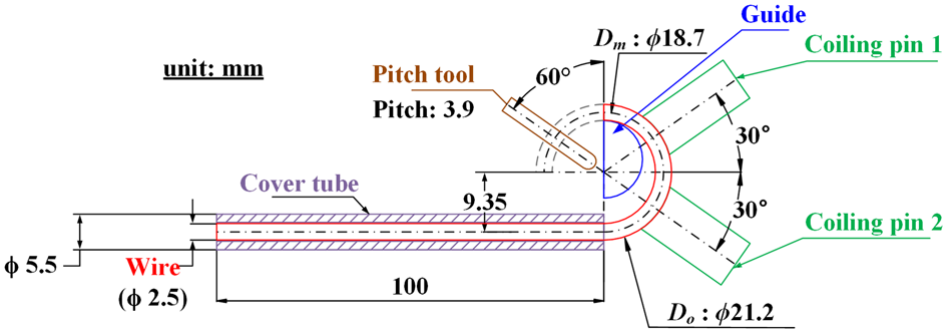
Schematic illustrating the cold coiling process.

Figure 10 depicts the cold coiling process. In this process, the OT wire is fed into the wire guide by the feed rolls. The wire guide feeds the wire and supports it to prevent bending during forming. The wire that passes through the wire guide is subjected to bending by the first and second coiling pins to form a helical spring with the desired inner diameter. The pitch of the spring is created by the transport of the pitch tool after one winding.

**Figure 11:**
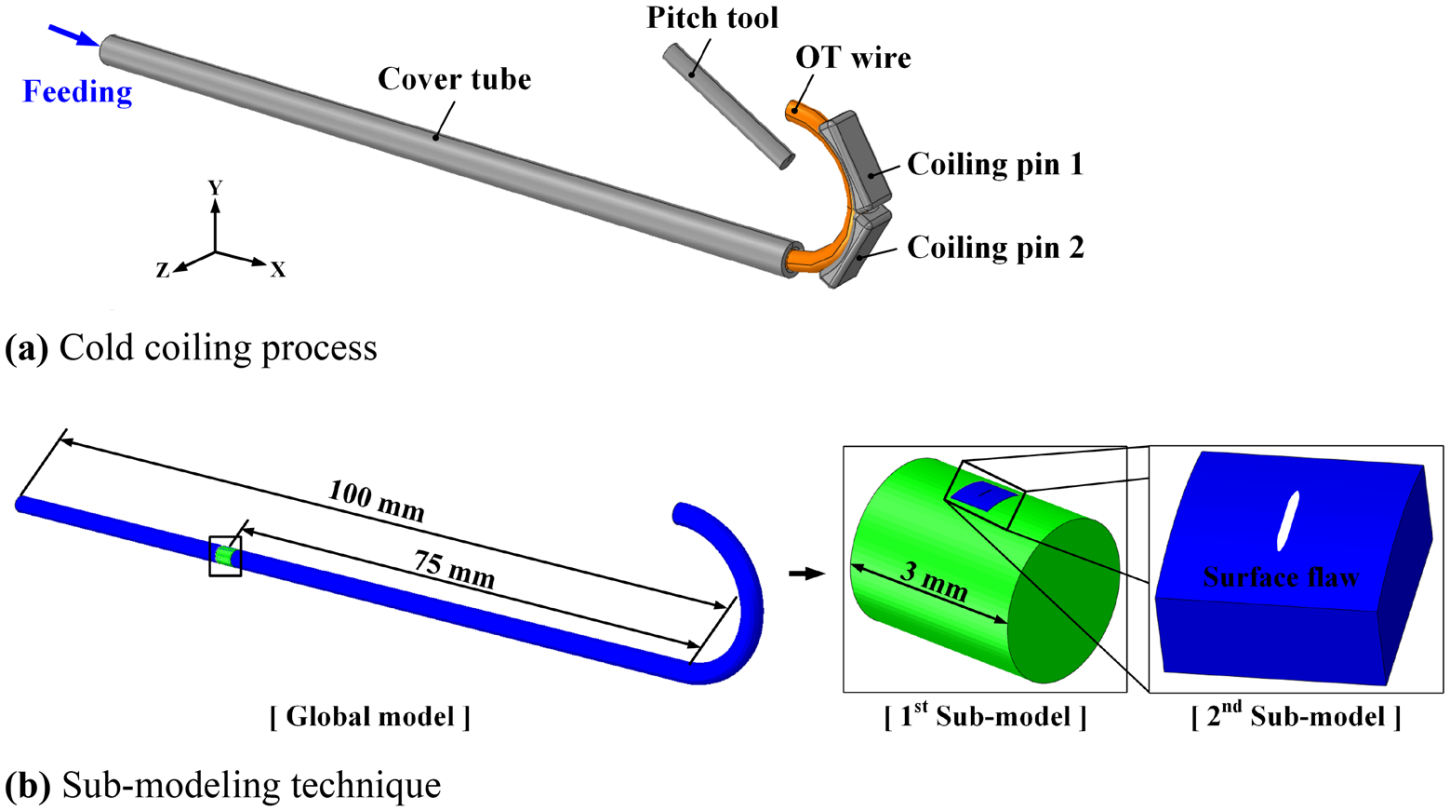
FE model of forming process for engine valve spring.

Figure 11a shows the FE model used to evaluate the change in the geometry of a surface flaw during the cold coiling process. The wire formation is mostly performed by the coiling pins, and the influence of the friction caused by the feed rolls is not significant because the oxide layer on the wire surface acts as a lubricant. Therefore, the feed rolls and the wire guide were simplified to a cover tube in the analysis model. The friction coefficient between the OT wire and the forming tools was set to 0.05. Two-dimensional rigid body plane and the fixation conditions were applied to the left end of the wire so that it could be fed in the X-axis direction at the same speed as the feed speed (0.6 m/s) by the feed rolls. Figure 11b shows the sub-modeling technique for applying a fine flaw to the wire. Considering the size of a surface flaw, sub-modeling was applied twice for a surface flaw with a depth of 20 µm or higher, and thrice for that with a depth less than 20 µm. A surface flaw was applied to a section formed with a uniform pitch. In the global model of the spring, the straight section of the wire had a length of 100 mm. As for the first sub-model, sub-model 1 with a length of 3 mm was applied to the 75 mm longitudinal position of the global model. Three-dimensional (3D) hexahedral eight-node elements were used in this simulation. In the global model and sub-model 1, the minimum edge length of each element is equal to 0.5 and 0.2 mm, respectively. After analyzing sub-model 1, a surface flaw was applied to sub-model 2. The length and width of sub-model 2 were three times the length of the surface flaw, to eliminate the influence of the boundary conditions of the sub-model; further, 50% of the length and width were applied as the depth of the sub-model. In sub-model 2, the minimum edge length of each element is 0.005 mm. The defined surface flaws were applied to the FE analysis, as shown in Table 3.

**Figure 12:**
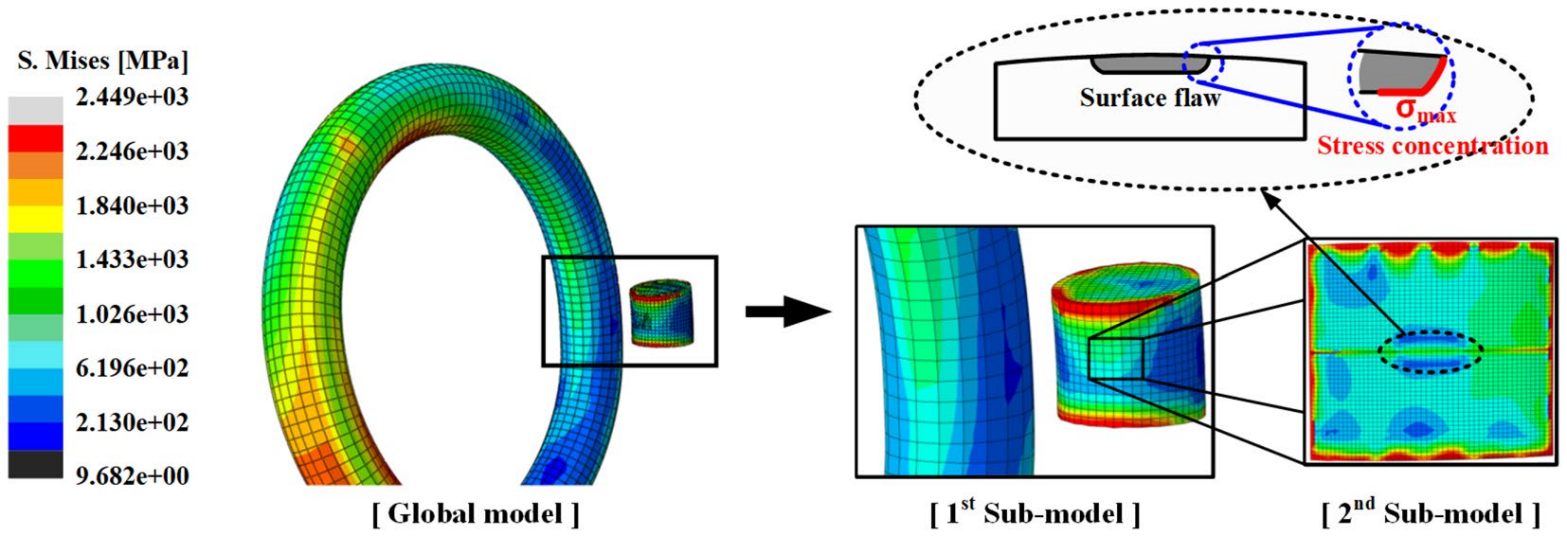
Effective stress distribution during the cold coiling process.

Figure 12 shows the stress distribution of the surface crack after the cold coiling process. The global model and sub-model 1 exhibited almost similar stresses of 1076 and 1079 MPa at the same position, thereby validating the sub-modeling technique. The local stress concentration occurred at the boundary edge of the sub-model. This appears to be due to the boundary conditions of the sub-model^7^. Sub-model 2, to which a surface flaw was applied, exhibited stress of 2449 MPa at the tip of the flaw during the cold coiling process due to stress concentration. As shown in Table 3, the surface flaws determined using the response surface method were applied to the inside of the spring. The FE analysis results indicated that no fracture occurred in the 13 surface flaw cases.

**Figure 13:**
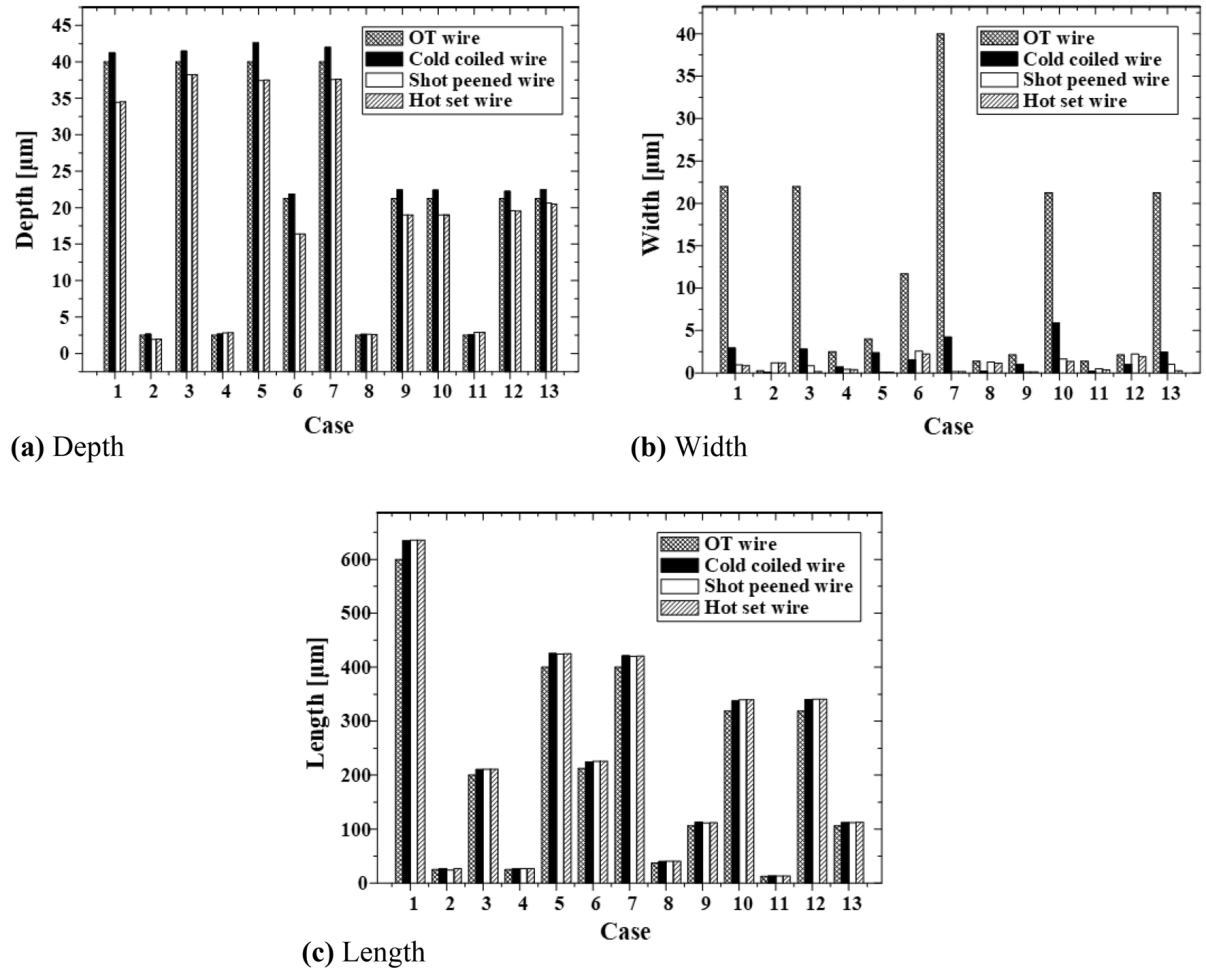
Deformation by depth, width, and length of the surface flaw on the OT wire during the manufacturing process.

During the coiling process among all the manufacturing processes, the depth of the surface flaw inside the spring increased by 0.1–2.62 µm (Fig. 13a) and the width decreased by 1.8–35.79 µm (Fig. 13b); further, the length increased by 0.72–34.47 µm (Fig. 13c). Because the transverse V-type flaw was closed in the width direction due to bending during the cold coiling process, it deformed into a V-type flaw with a steeper slope than the initial flaw.

The surface flaws were applied to the outside of the spring, and the probability of fracture during the cold coiling process was predicted through FE analysis. There is no probability of fracture of the outside surface flaw under the conditions listed in Table 3. In other words, no fracture occurred for surface flaw depths between 2.5 and 40 µm.

**Figure 14:**
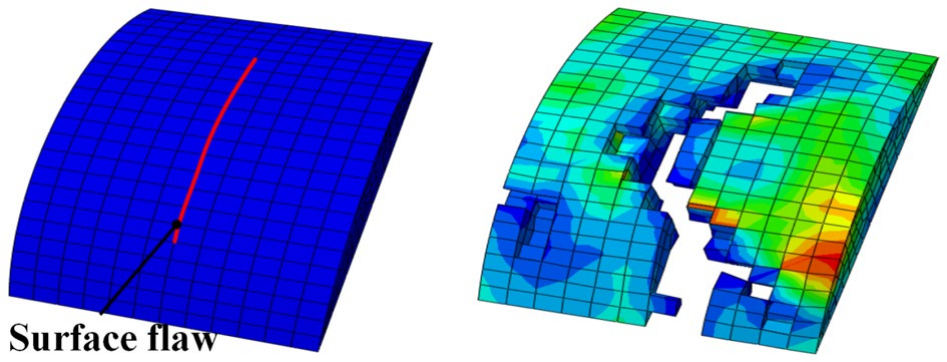
Spring fracture at a surface flaw depth of 55 µm.

To predict the critical surface flaw, the fracture on the outside was examined during the cold coiling process by sequentially increasing the flaw depth by 5 µm from 40 µm. Figure 14 shows the fracture at the surface flaw. A fracture occurred under the conditions having the depth (55 µm), width (2 µm), and length (733 µm). The depth of the critical surface flaw outside the spring was found to be 55 µm.

### Shot peening process

The shot peening process can inhibit crack propagation and improve fatigue life by generating compressive residual stress at a certain depth from the spring surface; however, it causes stress concentration by increasing the surface roughness of springs, thereby lowering the fatigue resistance of the springs. Therefore, a two-stage shot peening technique is used to manufacture high-strength springs, to complement the reduction in fatigue life due to an increase in surface roughness caused by shot peening. Two-stage shot peening can improve the surface roughness, maximum compressive residual stress, and surface compressive residual stress because a second shot peening is applied following the first shot peening^12–14^.

**Figure 15:**
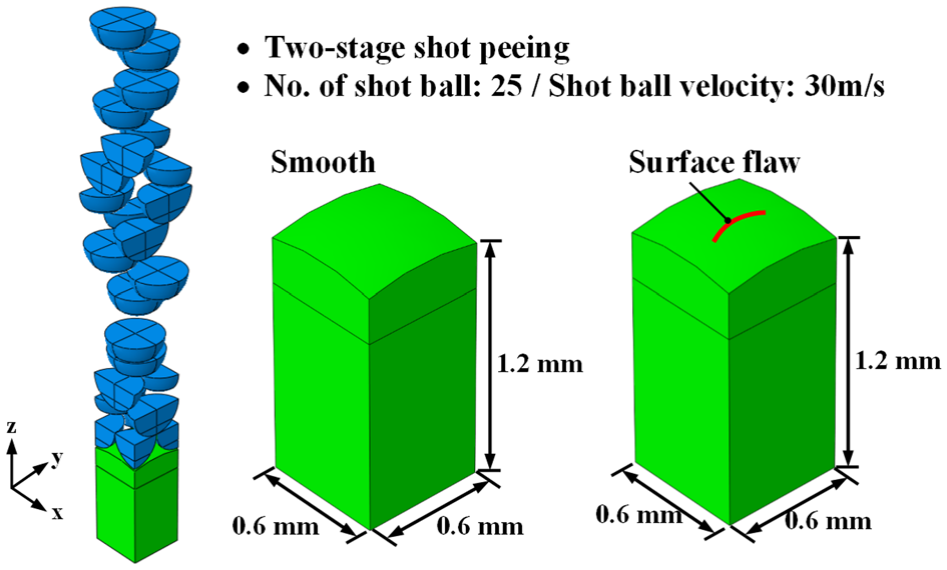
FE model for shot peening process.

Figure 15 shows the analysis model of the shot peening process. An elastoplastic model in which 25 shot balls were projected to the target local area of the OT wire for shot peening was set^15^. A surface flaw in the OT wire deformed by the cold coiling process was applied as the initial flaw in the shot peening analysis model. The residual stress resulting from the cold coiling process was removed by performing tempering before the shot peeing process. The following properties of the shot balls were used: density (ρ): 7800 kg/m^3^, elastic modulus (E): 210 GPa, and Poisson’s ratio (υ): 0.3. The friction coefficient between the shot balls and the material was set to 0.1. The shot balls with diameters of 0.6 and 0.3 mm were projected in the first and second shot peening processes at the same speed of 30 m/s. After the shot peening process (among other manufacturing processes shown in Fig. 13), the depth, width, and length of the surface flaw on the inside of the spring changed by − 6.79 to 0.28 µm, − 4.24 to 1.22 µm, and − 2.59 to 1.69 µm, respectively. The depth of the flaw was reduced by the plastic deformation caused by the shot balls projected vertically onto the material surface; particularly, the width of the flaw was reduced significantly. It appears that the closing of the flaw occurred because of the plastic deformation caused by shot peening.

### Hot setting process

In the hot setting process, the effects of cold setting and low-temperature annealing can be applied to the engine valve spring simultaneously. The cold setting maximizes the stress level of the spring by compressing it to the maximum possible level at room temperature. In this instance, if the stress of the engine valve spring is higher than the yielding point of the material, the engine valve spring is subjected to plastic deformation, thereby expanding the yielding point. Deflection occurs in the valve spring after plastic deformation, but the expanded yielding point ensures elasticity during actual valve spring operation. Low-temperature annealing increases the heat resistance and deformation resistance of valve springs under operation in high-temperature environments^2^.

The surface flaw deformed by the shot peening process in FE analysis and residual stress field measured by X-ray diffraction (XRD) equipment was applied to sub-model 2 (Fig. 8) to derive changes in the flaw by the hot setting process. The spring is designed to operate within the elastic range and was compressed from its free height of 50.5 mm to its solid height of 21.8 mm and then allowed to return to the initial height of 50.5 mm, as an analysis condition. The geometry of the flaw changed to a small extent during the hot setting process. It appears that the compressive residual stress of 800 MPa or more caused by shot peening inhibited the deformation of the surface flaw. After the hot setting process (Fig. 13), the depth, width, and length of the surface flaw changed by − 0.13 to 0.08 µm, − 0.75 to 0 µm, and 0.01–2.4 µm, respectively.

**Figure 16:**
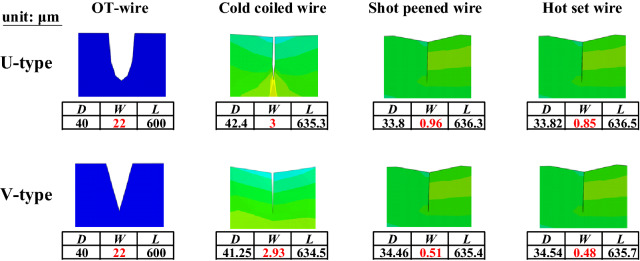
Deformation of U- and V-type surface flaws during the manufacturing process.

Figure 16 compares the deformation of the U- and V-type flaws having the same depth (40 µm), width (22 µm), and length (600 µm). The U- and V-type flaws exhibited greater changes in width than in length due to the closing in the width direction by the cold coiling process and shot peening. The V-type flaw developed a relatively higher depth and a steeper slope than the U-type flaw, indicating that a conservative approach is possible when the V-type flaw is applied.

In this section, the deformation of the initial flaw in the OT wire in each valve spring manufacturing process was discussed. The initial flaw in the OT wire was applied to the inside of the valve spring where the fracture was expected due to high stress during the spring operation. The V-type surface flaw in the OT wire in the transverse direction exhibited a slight increase in depth and length and a sharp decrease in width due to bending during the cold coiling process. The closing in the width direction occurred during the shot peening process, and there was little or insignificant deformation of the flaw during the final hot setting process. Large deformation occurred in the width direction during the cold coiling and shot peening processes, which involved plastic deformation. The V-type flaw inside the valve spring transformed into a T-type flaw due to the closing in the width direction during the cold coiling process.

## Residual stress distribution after each valve spring manufacturing process

In this section, the residual stress is measured, and FE analysis is performed for the shot peening and hot setting processes, which most greatly influence the fatigue life improvement for valve springs. The residual stress generated by two-stage shot peening is predicted and verified by comparing the measured residual stress of the OT wire subjected to two-stage shot peening with the FE analysis results. Furthermore, the residual stress in the final spring after the hot setting process is measured and applied to the analysis of the spring strength.

### Shot peening process

**Figure 17:**
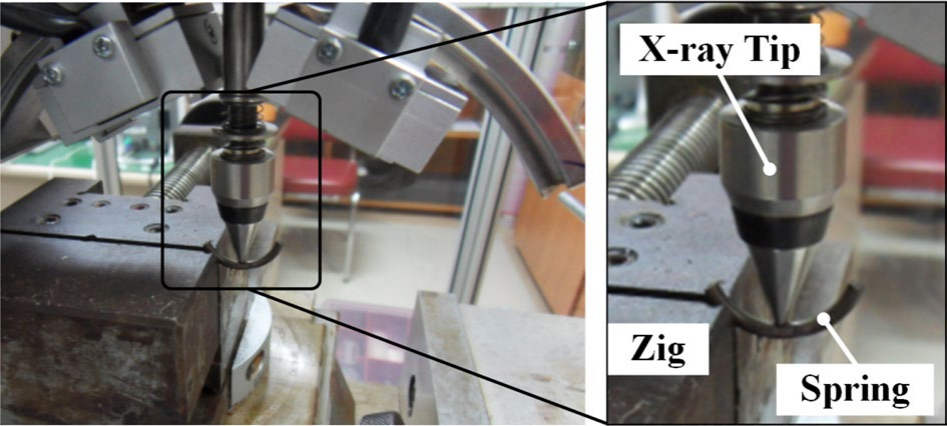
XRD equipment with engine valve spring specimen.

The residual stress inside the shot-peened valve spring was measured using XRD equipment (Xstress 3000). Figure 17 shows the spring and the XRD machine used for the measurement. To measure the residual stress of the valve spring after the two-stage shot peening process, wire cut electrical discharge machining (EDM) was applied to the 4th winding of the total 8.1 windings of the valve spring (by a length of 24 mm) to minimize the change in residual stress by cutting. The residual stress by depth was measured under the conditions listed in Table 4 after applying electropolishing at the depths of 0.03, 0.1, 0.14, and 0.19 mm. The prepared specimens are three for each polishing depth.

**Table 4:** Measurement conditions and parameters of the XRD machine.

Parameter	Value
System	XSTRESS 3000, stresstech Oy
Radiation	CrKa
Spot size	1 mm collimator
Exp. time	30 s, 5/5 tilts, − 40°/+ 40° psi angle, 5° psi oscillation
2θ	156.4°
Measure method	$d({\mathrm{sin}}^{2}\psi )$

**Figure 18:**
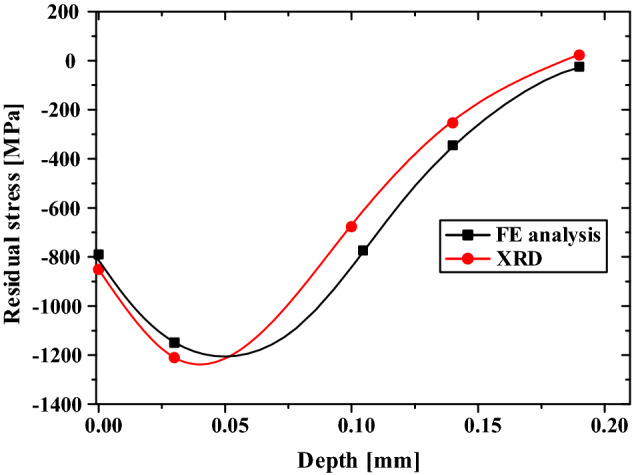
Residual stress profile of engine valve spring after two-stage shot peening.

To predict the residual stress in the engine valve spring by two-stage shot peening, FE analysis was performed on the model without surface flaws (Fig. 15). Figure 18 shows the residual stress measurement and FE analysis result. The results were generally similar, and the maximum compressive residual stress was found to be − 1200 to − 1250 MPa at depths of 0.03–0.04 mm. For flaw depths lower than the maximum flaw depth (40 µm) set in this study, the compressive residual stress was − 845.6 to − 1250 MPa. Such residual stress values are expected to inhibit the propagation of surface flaws. In the 0.05–0.15 mm depth range, the compressive residual stress decreased as the depth increased. Therefore, when the depth is greater than 0.05 mm, the inhibition of flaw propagation by the compressive residual stress is expected to be lowered. The compressive residual stress in the valve spring was predicted using the two-stage shot peening analysis technique and validated through XRD measurements.

### Hot setting process

**Figure 19:**
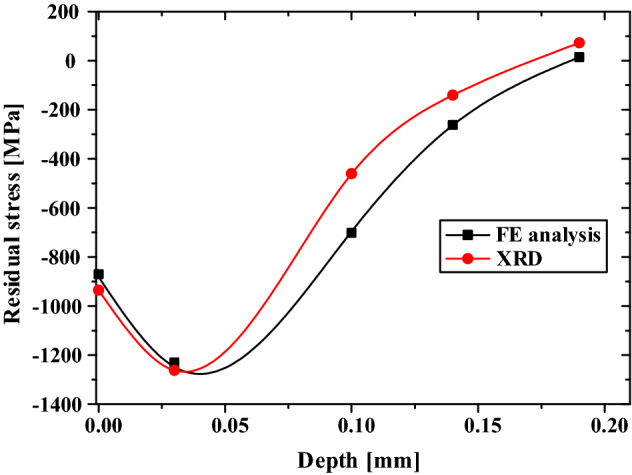
Residual stress profile of engine valve spring after hot setting.

The stress state existing on the surface of the valve spring after all the manufacturing process is required as the initial input condition for deriving the stress acting on the spring by conducting a strength analysis. Therefore, the residual stress in the final valve spring after all the spring manufacturing processes was measured to accurately identify the stress in the spring under operation. For measurement, wire cut EDM was applied to the 4th winding of the total 8.1 windings of the spring (by a length of 24 mm). To measure the residual stress, electro-polishing was applied at the depths of 0.03, 0.1, 0.14, and 0.19 mm. The residual stress was measured under the conditions listed in Table 4; Fig. 19 shows the residual stress distribution of the valve spring by depth. The compressive residual stress in the final spring was − 1194.6 MPa at a depth of 0.03 mm. Although this value is 5.5–55.4 MPa lower than the residual stress in the valve spring after shot peening, the change in stress by hot setting was evaluated to be insignificant.

**Figure 20:**
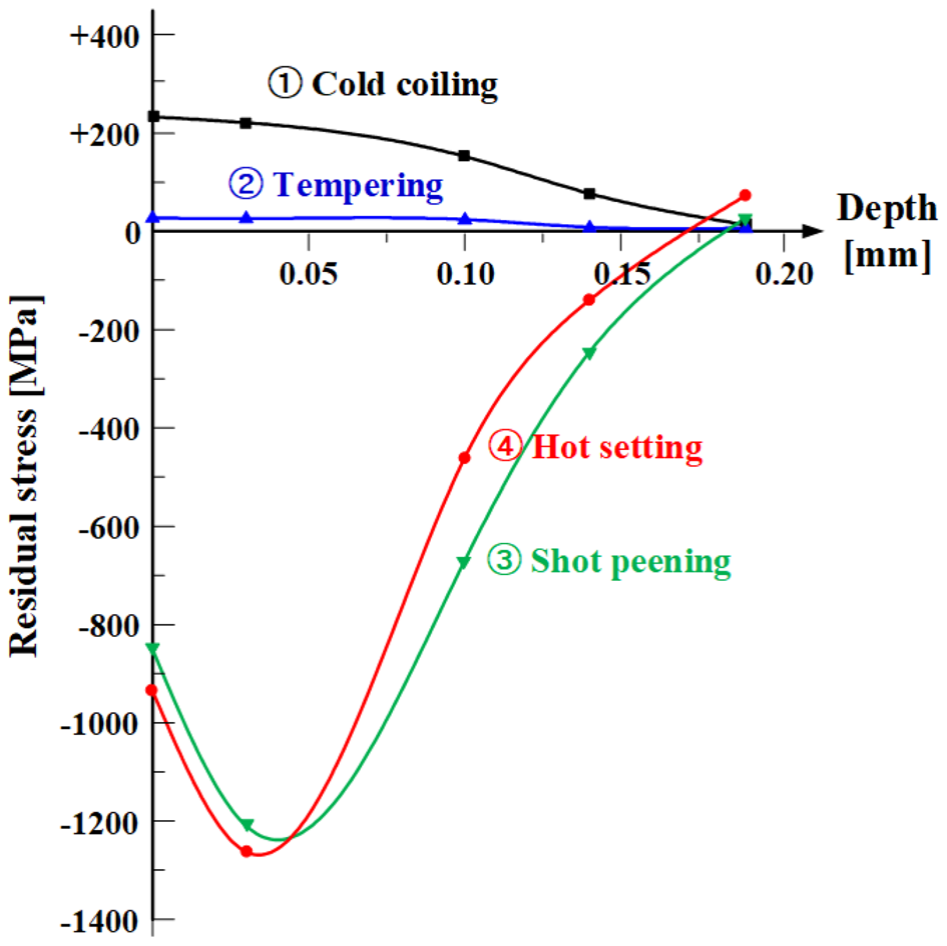
Residual stress and depth profile of the inner surface layer of cold-formed spring after each spring manufacturing process.

Figure 20 shows the residual stress in the OT wire after each spring manufacturing process. During the cold coiling process, the OT wire is subjected to bending by forming tools. In this instance, compressive stress acts on the inside of the spring, whereas tensile stress acts on the outside. After the cold coiling process, tensile and compressive residual stresses are generated on the inside and outside, respectively, of the spring. Because stress concentration occurs inside the valve spring during operation, the tensile residual stress generated after the cold coiling process affects the fatigue life of the spring adversely. In the tempering process, most of the residual stress generated during cold coiling is removed.

In the case of OT wires, the general tempering temperature is 360–460 °C, and the tempering process requires 20–30 min. After the tempering process, the shot peening process is performed to improve the surface hardness of the spring and to apply compressive residual stress. Then, the hot setting process is applied to prevent the deflection of the spring during operation and improve its fatigue life. In this process, the wire is subjected to torsion by an external force, and residual stress in the opposite direction is generated after the external force is removed. The hot setting process can improve the fatigue life of the spring because the generated residual stress acts in the opposite direction to the stress that acts during operation^16,17^.

## Fatigue life prediction of automotive engine valve spring

### Spring strength analysis

Wahl^18^ proposed spring-modifying factors, which have been widely used in design calculation equations. When the spring modifying factors are applied to a spring, the highest shear stress is found on the inside of the spring. Thus, the fracture of actual springs mainly occurs on the inside. Wahl’s theory excludes the effect of the pitch angle on compression coil springs and assumes that loads act at the coil center in the axial direction. The stresses acting on coil springs are predicted using FE analysis of late^19,20^. The application of FE analysis to coil spring strength analysis can reduce errors caused by the simplified calculation formula and improve the accuracy of the results.

**Figure 21:**
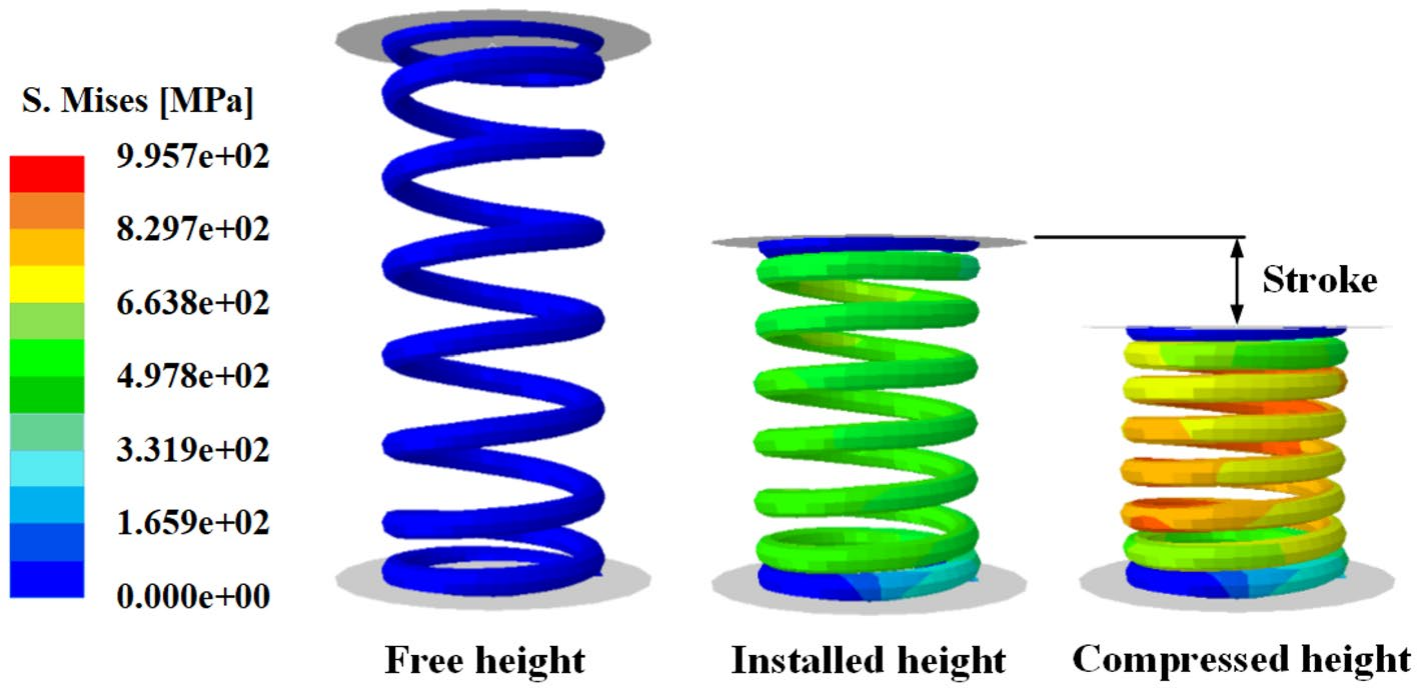
Operation conditions of automotive engine valve spring.

In this chapter, the strength of the valve spring is analyzed to evaluate the effects of compressive residual stress and surface flaws. The analysis was performed under the same conditions as the operating conditions of the actual spring. Figure 21 shows the analysis model.

The valve spring has a free height of 50.5 mm before being installed in an engine. During the engine operation, the spring operates at an installed height of 32 mm and a compressed height of 23.8 mm; at these heights, the spring is subjected to compression loads of 175 and 270 N, respectively. By substituting these values in Eq. (3)^7^, which applies the stress concentration factor of Wahl, the maximum shear stress acting on the inside of the spring was calculated to be 637.3 and 979.7 MPa at the installed height and compressed height, respectively.

3 $${\tau }_{max}=\frac{16PR}{\pi \cdot {d}^{3}}\left[\frac{4C-1}{4C-4}+\frac{0.615}{C}\right],$$

where *P*, *R*, *d*, and *C* denote the applied load, average radius of the spring, diameter of the wire, and spring index, respectively. Then, three cases, one without surface residual stress, one with surface residual stress, and another with a surface flaw were evaluated. First, the theoretical values and the shear stress of the model without residual stress were compared. The FE analysis results revealed 648.1 and 982.6 MPa at the installed height and compressed height, respectively; these values deviated from the theoretical values by ~ 1.6%. This appears to be because Wahl’s theoretical formula assumes only pure torsion of the spring and excludes the effect of the pitch angle.

The surface residual stress in the valve spring measured using the XRD machine was applied to the model with surface residual stress as the initial condition. Table 5 shows the maximum shear stress obtained by strength analysis under operation conditions. The maximum shear stress was found to be 516.3 and 822.4 MPa at the installed height and compressed height, respectively. The shear stresses were reduced by 15.2% and 17.7% at the compressed height and installed height, respectively, compared with those of the model without surface flaws. This appears to be because the compressive residual stress offsets the applied load during the valve spring operation.

**Table 5:** Maximum shear stress obtained by strength analysis of automotive engine valve spring with a surface flaw.

No. of case	Depth (*h*)	Width (*w*)	Length (*l*)	Aspect ratio ($\alpha$)	Length ratio ($\beta$)	Maximum shear stress (MPa)	
						Installed height	Compressed height
0	Without surface flaw					516.3	822.4
1	40	22	600	0.55	15	563.1	923.3
2	2.5	0.25	25	0.1	10	524.4	827.7
3	40	22	200	0.55	5	524.3	877.7
4	2.5	2.5	25	1	10	529.5	844.3
5	40	4	400	0.1	10	555.1	865.9
6	21.25	11.69	212.5	0.55	10	529.7	849.1
7	40	40	400	1	10	547.2	873.8
8	2.5	1.38	37.5	0.55	15	535.4	846.2
9	21.25	2.13	106.25	0.1	5	548.8	865.5
10	21.25	21.25	318.75	1	15	521.3	845.7
11	2.5	1.38	12.5	0.55	5	517.8	835.7
12	21.25	2.13	318.75	0.1	15	526.5	852.1
13	21.25	21.25	106.25	1	5	531.0	852.6

As for the model with a surface flaw, the surface residual stress measured from the valve spring and the flaw deformed during the spring manufacturing processes were applied as the initial conditions. The analysis results showed that the maximum shear stress occurred at the tip of the flaw of the spring. Compared with that of the model with residual stress and without surface flaws, the shear stress increased by 0.64–12.3% and 0.27–9.06% at the compressed height and installed height, respectively. When the compressive residual stress and surface flaw were present, the compressive residual stress reduced the applied stress, but the stress concentration at the surface flaw increased the stress level.

### Fatigue test

Automobile and spring manufacturers apply a fatigue life of greater than $5.5\times 1{0}^{7}$ cycles for valve springs, which requires a long service life. Spring manufacturers conduct durability tests under spring operating conditions to determine whether the targeted fatigue life is achieved from a quality control perspective. Owing to the structural nature of valve springs, the stress applied to them is limited. Further, owing to the application of the safety factor during the design of valve springs, valve springs do not fracture when the stress applied to them is similar to or lower than the fatigue strength; thus, deriving an S–N curve is difficult. Therefore, to derive the S–N curve of the valve spring, the stress acting on the spring was converted into fully reversed loading acting on the OT wire by applying the von Mises (Eq. (4)) and Goodman equations (Eq. (5)).

4 $$\tau =\frac{\sigma }{\sqrt{3}},$$

5 $$\frac{{\sigma }_{a}}{{S}_{e}}+\frac{{\sigma }_{m}}{{S}_{U}}=1.$$

Shear stress occurs on the inside of the spring during operation; thus, the shear stress was converted into equivalent stress, *σ*_*e*_, by applying the yielding condition of von Mises and then expressed as a fully reversed stress state with a stress ratio (*R*) of − 1 by applying the Goodman equation. The fatigue life of the valve spring can be predicted by deriving the S–N curve after conducting a rotary bending fatigue test with an OT wire.

**Figure 22:**
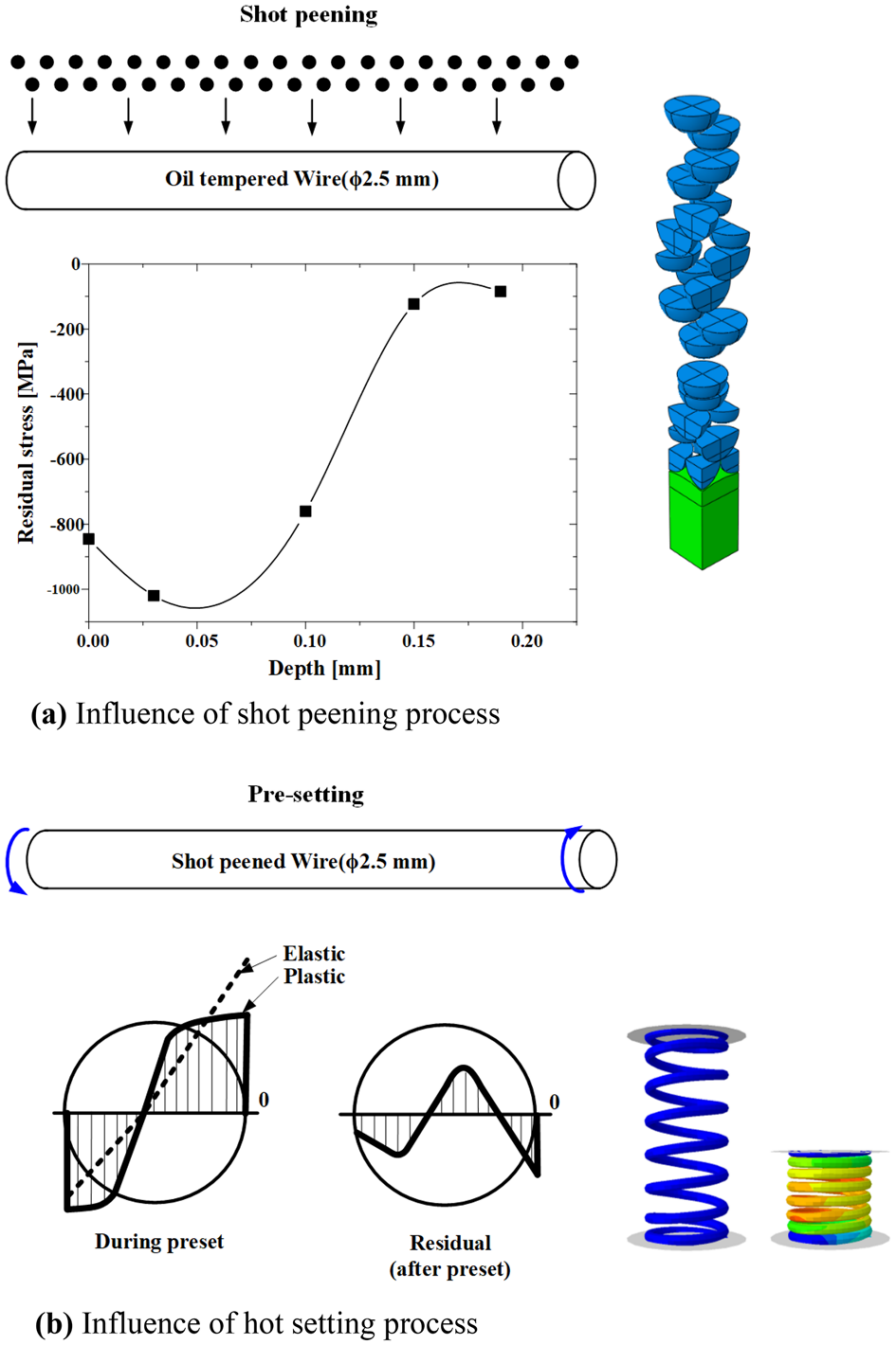
Application of shot peening and torsion to the OT wire.

The specimens used in the rotary bending fatigue test differed in size, surface roughness, and residual stress from the springs produced through the coiling, tempering, shot peening, and hot setting processes. Therefore, modifying factors must be applied to compensate for these differences; however, this would make the accurate prediction of the fatigue strength of a spring difficult, but a rough prediction is possible. Figure 22 shows the methods for compensating for the application of modifying factors. The specimens used in the rotary bending fatigue test were made of the same OT wire used for the valve spring. To generate the same residual stress as in the valve spring, an OT wire of length 670 mm was subjected to two-stage shot peening, which was the same as the shot peening process for the valve spring, as shown in Fig. 22a.

**Figure 23:**
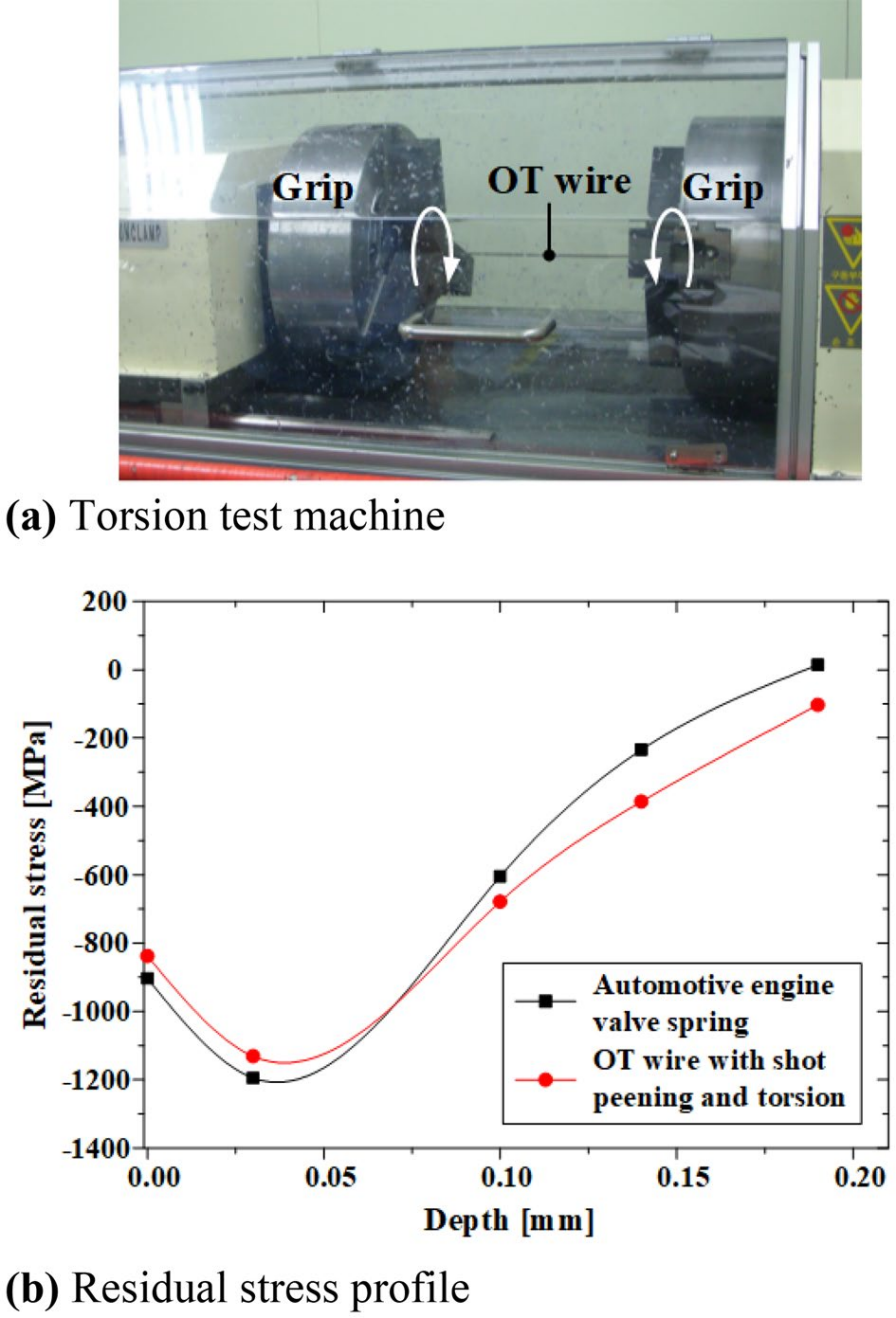
Comparison of residual stresses in automotive engine valve spring and OT wire.

It was assumed that pure torsion acts on the springs in the hot setting process (Fig. 22b), and the following equation was applied to provide the same effect to the OT wire.

6 $$\theta =\frac{2{\tau }_{max}}{Gd},$$

where *θ*, *τ*_*max*_, *G*, and *d* represent the unit torsion angle, maximum shear stress, shear modulus, and diameter of the wire, respectively. In the hot setting process, if 1116.9 MPa, 81.5 GPa, and 2.5 mm were applied as the maximum shear stress, shear modulus, and wire diameter, respectively, the torsion angle per unit length was calculated to be 0.628°/mm. The shot-peened 670 mm OT wire was twisted to a torsion angle of 420.7° at 5 rpm using a torsion test machine (Fig. 23A), and then allowed to return to its original position.

To validate this method, the residual stress in the OT wire subjected to shot peening and torsion was compared with that of the valve spring (Fig. 23b). The valve spring and OT wire exhibited compressive residual stresses of − 838.5 and − 903.4 MPa, respectively, on the surface; further, they showed similar maximum compressive residual stresses (− 1194.6 and − 1131.4 MPa, respectively) at a 0.03 mm depth. Therefore, the suitability of the application of shot peening and torsion to induce residual stress similar to that in the valve spring to OT wire was verified.

**Figure 24:**
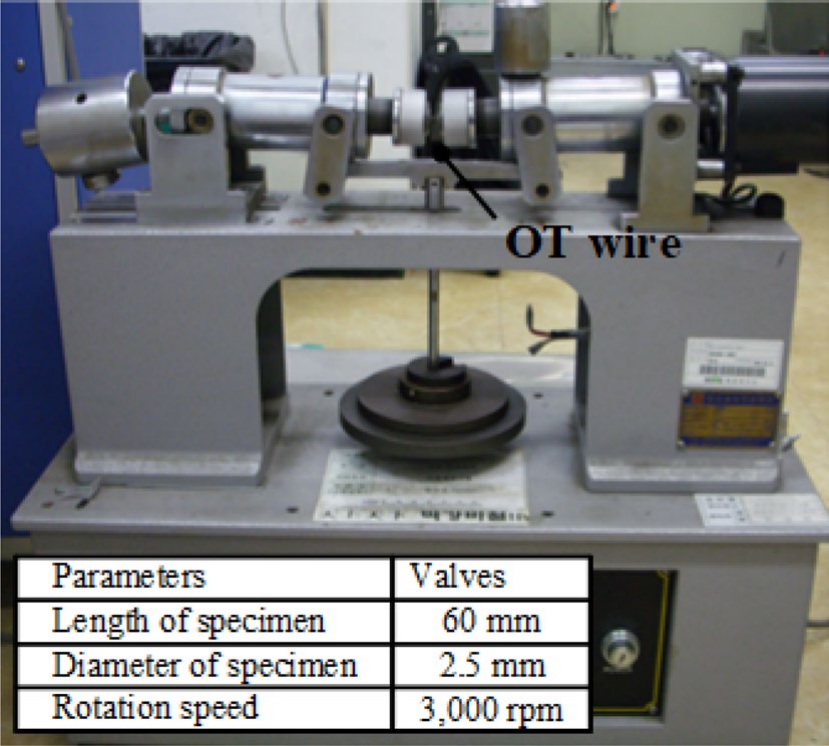
Rotary bending fatigue test machine.

The S–N curve of the shot-peened and twisted specimen was derived using a rotary bending fatigue test machine shown in Fig. 24. The dimension of the wire specimen was 2.5 mm in diameter and 60 mm in length, respectively. The ration speed is 3000 rpm.

### S–N curve application and life prediction

**Figure 25:**
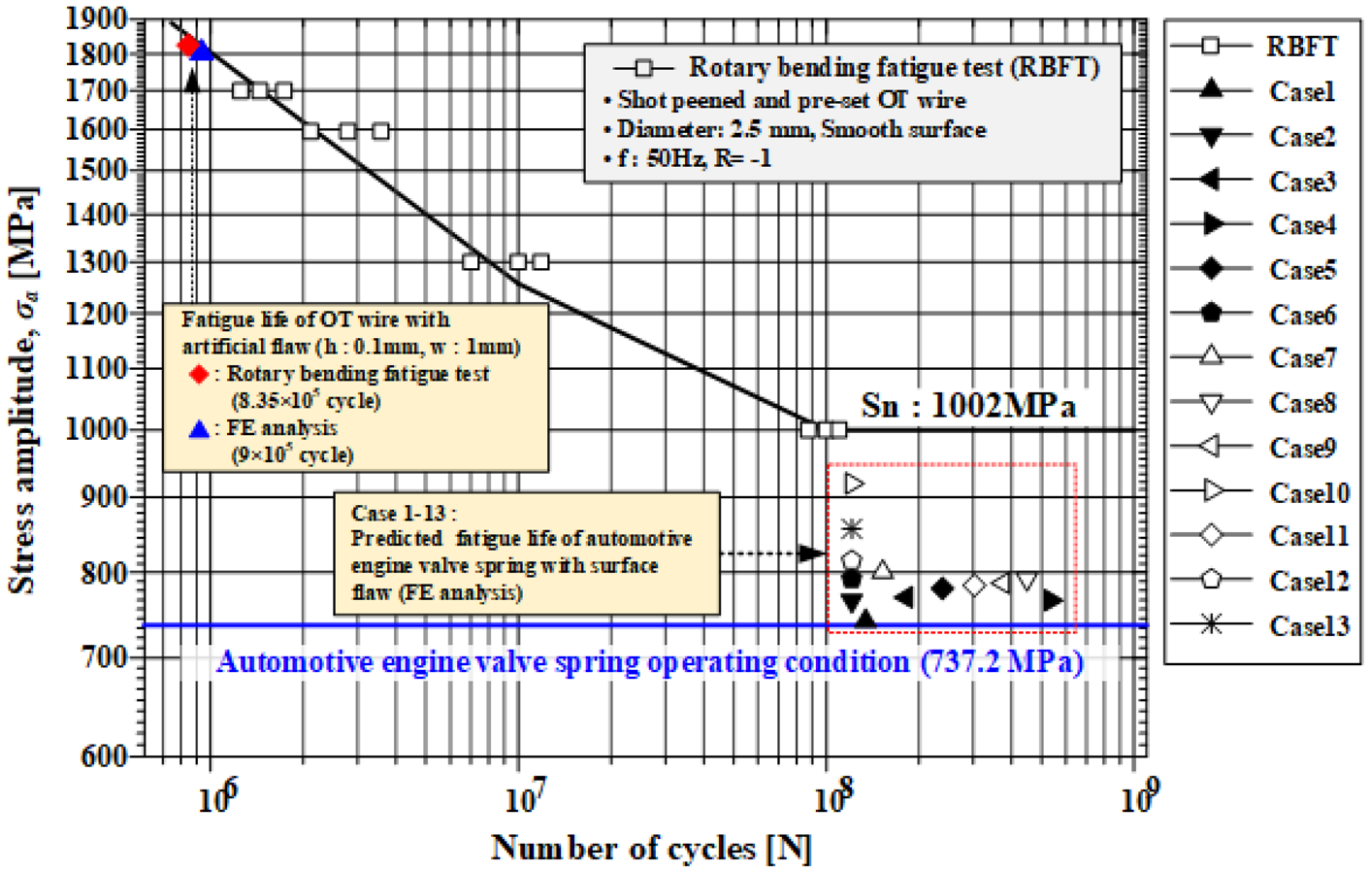
S–N curve for the shot peened and twisted OT wire.

**Figure 26:**
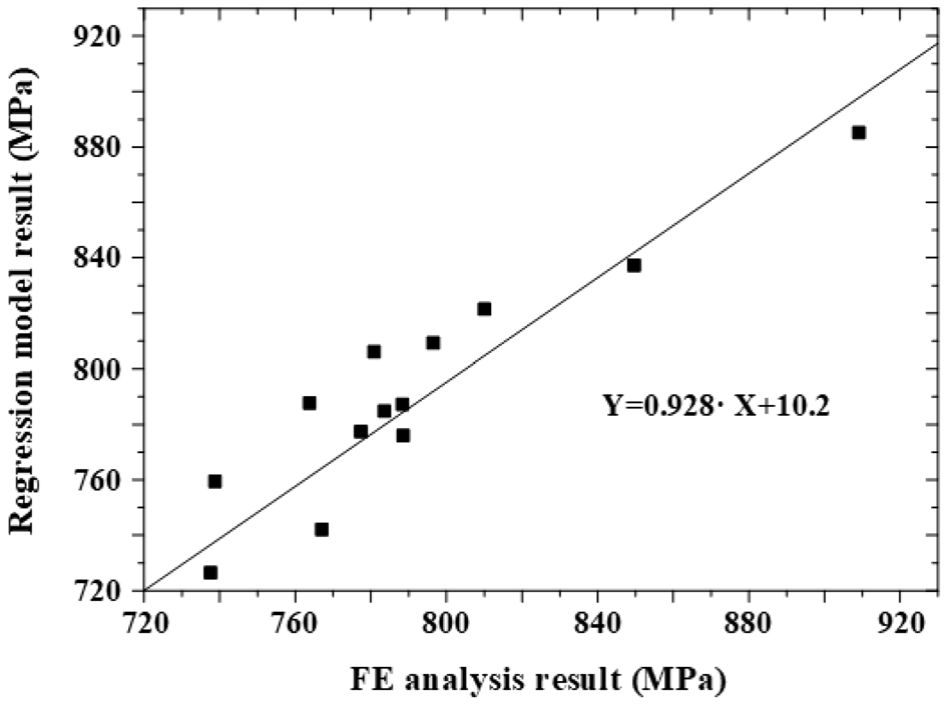
Comparison of the results of FE analysis and the regression model for predicting equivalent bending stress.

Figure 25 shows the S–N curve of the OT wire derived through the rotary bending fatigue test. To evaluate the effect of the surface flaw on the fatigue life of the spring, the strength analysis results of the surface flaw derived in subsection 6.1 were applied to the Goodman equation to obtain equivalent bending stress. The valve springs of cases 1 through 13 with an initial flaw depth of 40 µm or less exhibited a fatigue strength of 1002 MPa or less. Therefore, the fatigue life of the valve spring with a wire diameter of 2.5 mm, which is the target of this study, is expected to be more than 10^8^ cycles for surface flaws with a depth of 40 µm or less. A regression equation was derived by applying the response surface method to the initial flaws shown in Table 5:

7 $$Y=857.623-30.2671\cdot A+99.4419\cdot B-26.1820\cdot C+25.4924\cdot A2-87.8988\cdot B2+1.18837\cdot C2-0.175764\cdot A\cdot B+4.00201\cdot A\cdot C+1.45856\cdot B\cdot C,$$

where *Y*, *A*, *B*, and *C* denote the equivalent bending stress, flaw depth, aspect ratio, and length ratio, respectively. Figure 26 compares the results of Eq. (7) and those of FE analysis, from which similar stress distributions were observed. Using Eq. (7), the approximate equivalent bending stress can be derived using the flaw depth, aspect ratio, and length ratio.

**Figure 27:**
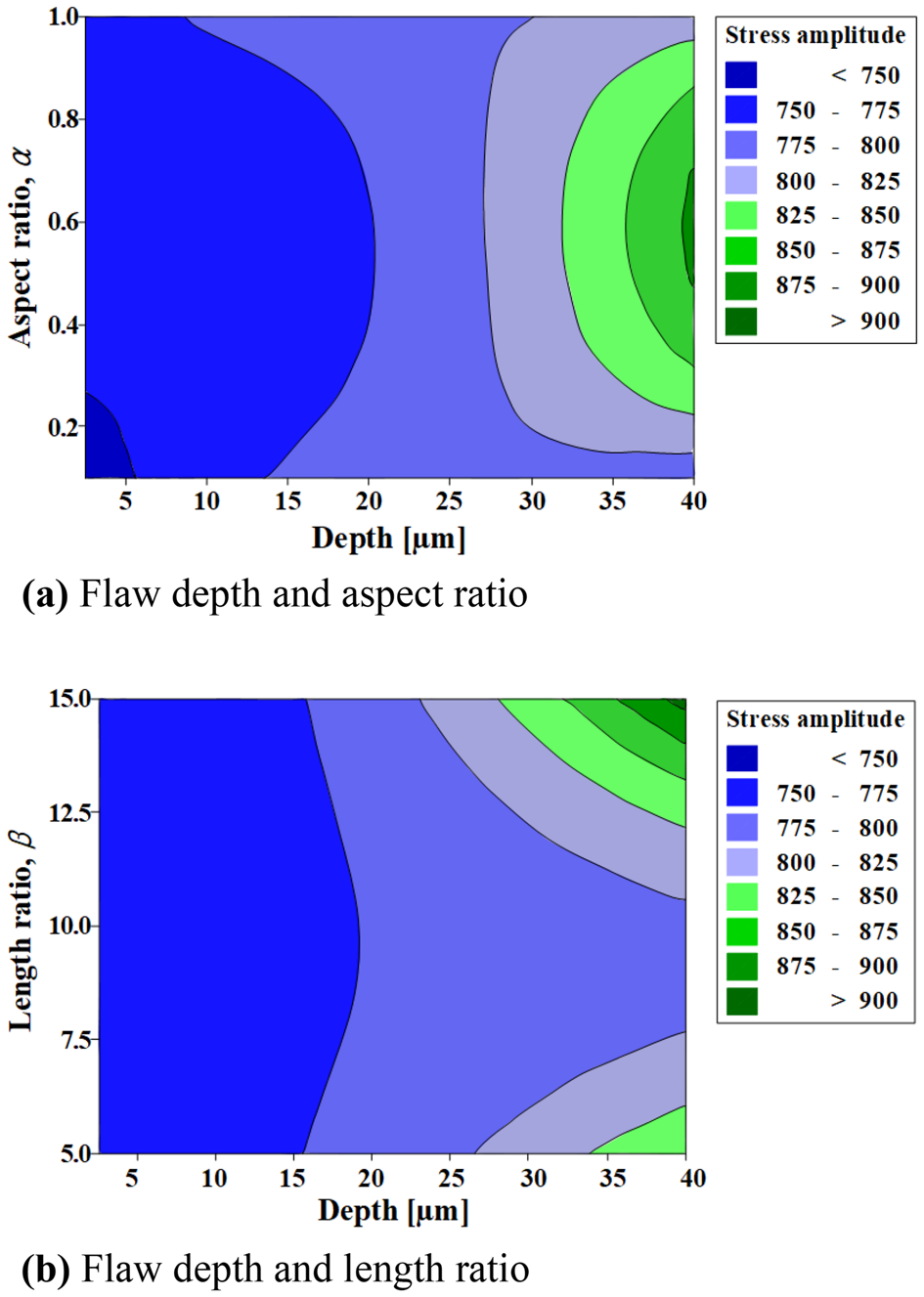
Influence of initial surface flaws on stress amplitude.

Figure 27 shows the relationships between the flaw depth, aspect ratio, length ratio, and equivalent bending stress. The flaw depth exhibited the highest influence on the equivalent bending stress, followed by the length ratio and aspect ratio. The equivalent bending stress increased as the flaw depth and length ratio increased (Fig. 27a), and the stress did not change significantly with respect to the aspect ratio (Fig. 27b). The influence of the aspect ratio on the equivalent bending stress was evaluated to be the lowest; this was because the initial aspect ratio changed to nearly 0 by the closing of the flaw in the width direction during the spring manufacturing processes. To predict the critical flaw, the aspect ratio with the lowest influence was set to 0.55, and the regression equation was applied for the surface flaw depth and length ratio ranges of 5–80 µm and 5–15, respectively.

**Figure 28:**
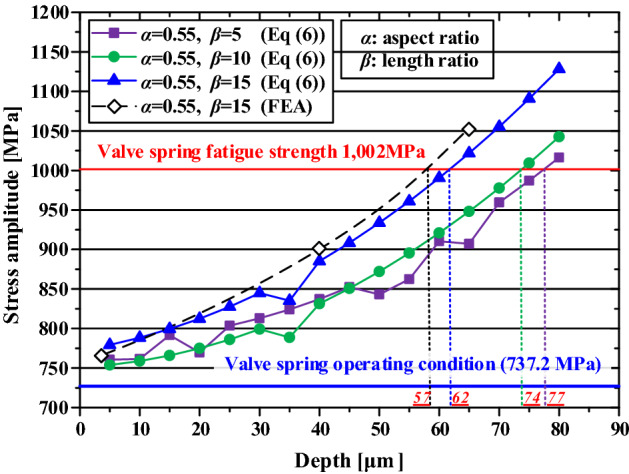
Depth–stress amplitude curve for predicting the critical flaw depth.

The equivalent bending stress increased as the flaw depth and length ratio increased (Fig. 28). Because the equivalent bending stress varies depending on the length ratio despite the same flaw depth, both the flaw depth and length ratio must be considered for the critical flaw. Using the regression equation, the critical flaw depths of 77, 74, and 62 µm were predicted for surface flaws with an aspect ratio of 0.55 and length ratios of 5, 10, and 15, respectively. To validate this method of predicting the critical flaw using the regression equation, FE analysis was performed for an aspect ratio of 0.55 and length ratio of 15, and the results were compared. The FE analysis predicted the critical flaw depth to be ~ 57 µm, a deviation of ~ 9% compared with that calculated by the regression equation. However, the trend line of FE analysis was similar to that of the regression equation, indicating that the critical flaw can be predicted using the regression equation.

**Figure 29:**
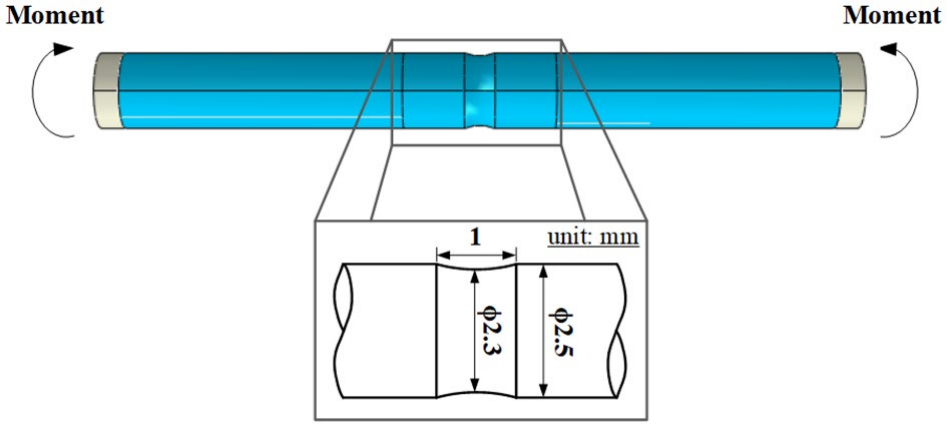
FE model of rotary bending fatigue analysis.

A rotary bending fatigue test was conducted using an OT wire with an artificial flaw to validate the method of predicting the fatigue life using strength analysis; then, the predicted fatigue life was compared with FE analysis results. Local chemical polishing was applied to the artificial flaw (depth: 0.1 mm, width: 1 mm) to minimize the change in residual stress caused by machining. In the rotary bending fatigue test, bending stress of 1151 MPa and rotation speed of 3000 rpm were applied. The rotary bending fatigue analysis was conducted using the analysis model shown in Fig. 29. The maximum and minimum stresses of 1456 and − 2217 MPa were generated by the artificial flaw. By substituting these values in the Goodman equation, the equivalent bending stress was calculated to be 1840.3 MPa and the fatigue life was predicted to be 9 × 10^5^ cycles. The fatigue life of the specimen with artificial flaw was evaluated to be 8.35 × 10^5^ cycles, which is similar to the predicted value (Fig. 25). This demonstrated the validity of the method of predicting the fatigue life of the valve spring using the stress level of a surface flaw through FE analysis.

## Conclusions

This study evaluated the influence of the depth of a surface flaw in an OT wire on the fatigue life of an automotive engine valve spring. The deformation of the surface flaw in the OT wire during the valve spring manufacturing processes was derived via FE analysis, and the residual stress of the final spring was measured and applied to the spring stress analysis model. The strength of the valve spring was analyzed to examine the presence of residual stress and compare the applied stress levels by the surface flaw. The influence of the surface flaw depth on the fatigue life of the spring was evaluated by applying the stress on the surface flaw derived through the spring strength analysis to the S–N curve derived through a rotary bending fatigue test. The results of this study are presented here.

1. The surface flaws in the OT wire were standardized to a V-type flaw. Strength analysis was performed by applying the flaw to the inside of the spring in the longitudinal, transverse, and oblique directions with respect to the axial direction of the wire. The highest stress was observed in the transverse direction.
2. The transverse V-type initial flaw in the OT wire deformed into a sharp V-type flaw because of the closing in the width direction on the inside of the spring during the cold coiling process. Owing to the plastic deformation caused by shot balls in the shot peening process, the depth decreased and closing in the width direction occurred, thereby deforming the flaw into a T-type flaw. In the hot setting process, the surface flaw changed by a small extent. The surface flaw in the OT wire was deformed in the cold coiling and shot peening processes.
3. The compressive residual stress by depth after the two-stage shot peening process, which is applied to ultrahigh-strength valve springs, was predicted through FE analysis, and the results were verified by comparison with residual stress measurement results. FE analysis was useful in predicting the compressive residual stress after the shot peening process. After the hot setting process, the residual stress in the final spring was measured and applied to the valve spring strength analysis.
4. The maximum shear stress of the spring with a surface flaw was derived through a spring strength analysis considered the residual stress of the entire manufacturing process, and the results were applied to the S–N curve to predict the fatigue life. The fatigue life prediction method was validated through a fatigue test using an OT wire with an artificial flaw and FE analysis.
5. The aspect ratio and length ratio of the surface flaw were used to evaluate the influence of the depth, width, and length of the surface flaw on the fatigue life of the valve spring. The flaw depth exhibited the greatest influence on the fatigue life, followed by the length ratio and aspect ratio. The equivalent bending stress increased as the flaw depth and length ratio increased. The aspect ratio had the lowest influence on the fatigue life due to the closing in the width direction during the spring manufacturing processes.
6. For flaws with a depth of less than 40 µm, which is the existing criterion for surface flaw management, the fatigue life did not reduce. The critical surface flaw depths that do not reduce the fatigue life were predicted to be 77, 74, and 62 µm for the length ratios of 5, 10, and 15, respectively.

## Data Availability

All data generated or analyzed during this study are included in this published article.
